# Development of a Disease‐Specific Virtual Malaria Population for Physiologically‐Based Pharmacokinetic Modeling

**DOI:** 10.1002/psp4.70294

**Published:** 2026-07-16

**Authors:** Junjie Ding, Qi Pei, Richard M. Hoglund, Joel Tarning

**Affiliations:** ^1^ Mahidol Oxford Tropical Medicine Research Unit, Faculty of Tropical Medicine Mahidol University Bangkok Thailand; ^2^ Centre for Tropical Medicine and Global Health, Nuffield Department of Medicine University of Oxford Oxford UK; ^3^ Department of Pharmacy, The Third Xiangya Hospital Central South University Changsha China; ^4^ Infectious Diseases Data Observatory Oxford UK

## Abstract

Malaria remains a major global health challenge, with an urgent need for new therapies to combat the evolving resistance to anti‐malarial treatments. Physiologically‐based pharmacokinetic (PBPK) modeling offers a promising approach to accurately predict PK, optimize dosing strategies, reduce development time and cost, and de‐risk the development of novel anti‐malarial compounds. In this study, we developed and validated a virtual malaria population, reflecting the pathophysiological changes associated with acute uncomplicated malaria infections. Key alterations included elevated plasma α1‐acid glycoprotein (+118%), reduced plasma albumin (−16.8%), decreased estimated glomerular filtration rate (−10%), reduced hepatic enzyme abundance (−26% to −42%), increased blood flow (+40%), and prolonged gastric emptying time (+~45 min), with parameter magnitudes systematically obtained from the literature. A dynamic function was incorporated to describe the evolution of these biological changes during the acute infection and treatment. The virtual population was used for PK predictions of quinine, dihydroartemisinin, amodiaquine, and desethylamodiaquine, which differ in metabolic pathways and plasma protein binding characteristics. Sensitivity analyses indicated that plasma protein levels had the largest impact on PK exposure, followed by enzyme abundance and blood flow, whereas eGFR contributed minimally. The developed virtual malaria population provides a proof‐of‐concept translational framework that may improve PK predictions during the acute infection and support the development of novel anti‐malarial therapies.

## Study Highlights

- What is the current knowledge on the topic?
  ○ Malaria remains a major global health challenge, with an urgent need for new therapies to combat the evolving resistance to anti‐malarial treatments. Malaria infection is associated with substantial physiological changes that can significantly affect the PK in patients. PBPK modeling incorporating a virtual malaria population has the potential to improve PK predictions in patients and to inform dose selection during novel anti‐malarial drug development.
- What question did this study address?
  ○ This study aimed to develop and validate a virtual population that reflects pathophysiological changes associated with acute uncomplicated malaria infection, including alterations in plasma protein, estimated glomerular filtration rate, reduced hepatic enzyme abundance, and blood flow.
- What does this study add to our knowledge?
  ○ The developed virtual malaria population was applied to several anti‐malarial drugs that differ in metabolic pathways and plasma protein binding characteristics and demonstrated overall improved predictive performance compared with a virtual healthy population.
- How might this change drug discovery, development, and/or therapeutics?
  ○ The developed virtual malaria population provides a proof‐of‐concept translational framework that may improve PK predictions during the acute infection and support the development of novel anti‐malarial therapies.

## 1 Introduction

Malaria remains a global health issue, with an estimated 282 million malaria cases and 610,000 malaria‐related deaths in 2024, according to WHO malaria report 2025 [1]. The current first‐line treatment of uncomplicated malaria is artemisinin‐based combination therapies (ACTs). However, resistance to artemisinin and partner drugs has developed in Southeast Asian [2, 3] and artemisinin resistance has now also emerged independently in eastern Africa [4, 5], posing a significant challenge for treatment of malaria. Indeed, unacceptably high levels of ACT treatment failures have been observed in the Greater Mekong Subregion [6, 7]. Strategies to tackle this emerging resistance include developing new treatment approaches based on existing drugs such as triple ACTs that combine one short‐acting artemisinin derivative with two long‐acting partner drugs, to extend the useful therapeutic life of existing anti‐malaria drugs [8, 9]. However, the current approaches are unable to radically resolve this problem; there remains a high unmet need for novel anti‐malarial drugs with diverse mechanisms of action. The global anti‐malarial drug pipeline shows that there are dozens of anti‐malaria candidate compounds currently in discovery and clinical development stages [10].

Pharmacokinetic‐pharmacodynamic (PK‐PD) modeling plays a crucial role in anti‐malarial drug discovery and development to identify and optimize drug doses to achieve therapeutic drug concentrations while minimizing safety risks. Approaches such as allometric scaling and physiologically‐based pharmacokinetic (PBPK) modeling have been developed to predict PK profiles of drugs in humans based on preclinical data, enabling accelerated and de‐risked drug development. The use of PBPK modeling approaches has become increasingly important, as it enables the integration of drug data and systems biology data, providing a more accurate prediction of human PK. PBPK models have been developed for a number of existing and novel anti‐malaria drugs, showing satisfactory predictive performance in virtual healthy populations [11]. However, substantial biological changes occur during a malaria infection, significantly affecting PK of anti‐malarial drugs, as observed with compounds like quinine [12], dihydroartemisinin (DHA) [13], and piperaquine (PQ) [14]. Directly extrapolating PK from healthy participants to malaria patients without accounting for the biological changes associated with malaria infection is problematic.

Biological changes associated with acute malaria infection can affect absorption, distribution, metabolism, and elimination processes of a drug. Specifically, this include reduced hepatic CYP450 enzyme activity due to pro‐inflammatory cytokines [15], slightly reduced GFR resulting from mild renal impairment [16], decreased plasma albumin level caused by inflammation [17], increased plasma α1‐acid glycoprotein (AGP) as a response to an acute infection [18], increased cardiac output and liver blood flow due to fever and systemic inflammation [19], and altered gut motility as a consequence of fever. These factors should be considered in PK prediction in malaria patients. Previous population PK analyses have demonstrated that malaria infection alters drug disposition compared with healthy volunteers, with reported changes across multiple PK parameters, including reduced clearance (e.g., amodiaquine [20]), reduced bioavailability (e.g., piperaquine [14]), and reduced volume of distribution (mefloquine [21]).

In this study, we aimed to develop a virtual malaria population by integrating malaria infection‐relevant biological changes into PBPK model predictions and verify the results by using observed PK data of several typical anti‐malarial drugs in patients with acute uncomplicated malaria infection. The developed virtual malaria population will provide a useful tool to more accurately predict drug PK properties in the target population and inform novel anti‐malaria drug development.

## 2 Methods

### 2.1 PBPK Model Framework

**FIGURE 1:**
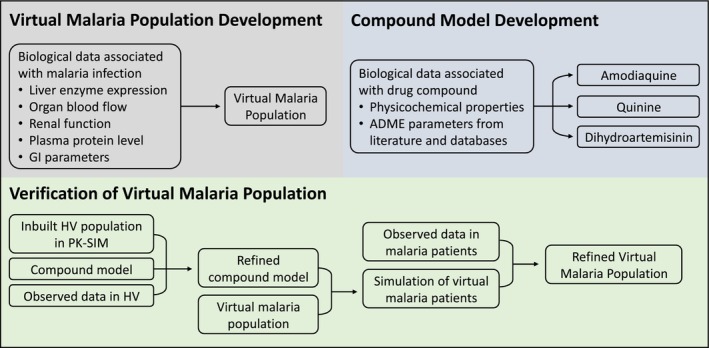
Flowchart of development of a virtual population with acute uncomplicated malaria.

The overall PBPK development framework for a virtual population with uncomplicated malaria infection is shown in Figure 1. Briefly, a literature review was first conducted to gather biological data associated with malaria infection. With these data, a virtual malaria population was developed in MoBi (version 11.0, Open Systems Pharmacology Suite, Bayer Technology Services, Leverkusen, Germany) based on a built‐in healthy population in the PK‐SIM software.

Compound models for several anti‐malarial drugs (i.e., amodiaquine, quinine, and DHA) were developed using their respective physiochemical data and ADME paraments. Compound models were verified and optimized based on observed PK data in healthy populations.

Finally, the virtual malaria population, along with compound models, was used to predict PK properties in patients with uncomplicated malaria infection. These predictions were verified using observed PK data in malaria patients, and models were refined if needed.

### 2.2 Literature Review

A broad literature review was conducted to obtain basic information regarding key biological changes associated with malaria infection. This information was reviewed, organized, and further discussed with internal malaria experts. After this, we conducted a narrower literature review to gather specific references associated with uncomplicated malaria infection. PubMed and Google Scholar were used together with search terms such as “uncomplicated malaria,” “non‐severe malaria,” “biological changes,” “pharmacokinetics,” “healthy subjects,” “plasma protein,” “blood flow,” “cardiac output,” “liver enzyme,” “renal function,” and “eGFR.” The timeframe of literature search was from inception to December 2024.

### 2.3 Anti‐Malarial Drug Selection for Compound Models

Since most anti‐malarial drugs are predominantly eliminated via hepatic pathways [11], the model drugs were selected to represent hepatically cleared compounds. The strategy for selecting anti‐malarial drugs for compound models included (1) primary metabolism via a single liver phase 1 or phase 2 enzyme, (2) protein binding primarily to plasma albumin or α1‐acid glycoprotein, (3) availability of longitudinal PK data in both healthy volunteers and patients with uncomplicated malaria infection (thus providing PK data in both the acute and convalescence phases of the infection).

According to the above strategy, we chose three anti‐malarial drugs to be used as compound models.

1. *Amodiaquine*: primarily metabolized by liver CYP2C8 with very high clearance and limited renal elimination, forming its active metabolite, *desethylamodiaquine*. Amodiaquine primarily binds to plasma albumin, while desethylamodiaquine binds both albumin and α1‐acid glycoprotein (AGP) at nearly the same fraction.
2. *Dihydroartemisinin*: primarily metabolized by liver phase 2 enzymes UGT1A9 and UGT2B7, with minimal renal elimination. Dihydroartemisinin binds primarily to AGP.
3. *Quinine*: primarily metabolized by liver CYP3A4, with minimal renal elimination (less than 20%). Quinine binds primarily to AGP.

The compound‐specific parameters in the PBPK models were collected from relevant published literature and were further optimized using observed PK data in healthy volunteers, if needed. PK data for selected anti‐malarial drugs were collected from published articles indexed in the PubMed database, and only studies reporting relevant PK profiles for the selected drugs were considered for inclusion.

### 2.4 Equation to Describe Biological Change Over Time

The disease course of acute uncomplicated malaria infection is relatively short, with patients typically recovering within a few days after receiving effective anti‐malarial treatment. However, the physiological changes associated with acute malaria might take longer to return to baseline, compared to simply eliminating the parasites. These dynamic processes were considered during the development of the virtual population.

In general, we utilized existing time‐varying relationships for physiological parameters when available from longitudinal studies. In cases where such data were absent or limited, we assumed that patients fully recover from malaria infection within 1 week of treatment, with significant alterations in biological parameters returning to normal levels. All dynamic and time‐varying changes, including the specific implementations, are specified for each physiological parameter in Table 1.

**TABLE 1:** Relative physiological changes associated with acute uncomplicated malaria infection.

Parameter	Malaria‐associated relative change	Reference	Equation
Hepatic enzyme clearance			
CYP2C8	↓31%	[15]	$\mathrm{CYP}2C8\left(t\right)=0.69+0.31\times \left(1-e^{-\frac{0.693\times t}{23}}\right)$
CYP2B6	↓42%	[15]	$\mathrm{CYP}2B6\left(t\right)=0.58+0.42\times \left(1-e^{-\frac{0.693\times t}{23}}\right)$
CYP2C9	↓31%	[15]	$\mathrm{CYP}2C9\left(t\right)=0.69+0.31\times \left(1-e^{-\frac{0.693\times t}{104}}\right)$
CYP2C19	↓31%	[15]	$\mathrm{CYP}2C19\left(t\right)=0.69+0.31\times \left(1-e^{-\frac{0.693\times t}{26}}\right)$
CYP2D6	↓41%	[15]	$\mathrm{CYP}2D6\left(t\right)=0.59+0.41\times \left(1-e^{-\frac{0.693\times t}{70}}\right)$
CYP2E1	↓26%	[15]	$\mathrm{CYP}2E1\left(t\right)=0.74+0.26\times \left(1-e^{-\frac{0.693\times t}{27}}\right)$
CYP3A4	↓42%	[15]	$\mathrm{CYP}3A4\left(t\right)=0.58+0.42\times \left(1-e^{-\frac{0.693\times t}{79}}\right)$
UGT	↓27%	[15]	$\mathrm{UGT}\left(t\right)=0.73+0.27\times \left(1-e^{-\frac{0.693\times t}{24}}\right)$
Renal clearance			
GFR	↓10%	[16]	$\mathrm{GFR}\left(t\right)=0.9+0.1\times \left(1-e^{-0.0274\times t}\right)$
Protein binding			
Albumin level	16.8%↓	[17]	$\mathrm{ALB}\;\left(t\right)=0.832+0.168\times \left(1-e^{-0.00640\times t}\right)$
Alpha‐1‐acid glycoprotein	↑118%	[18]	$\mathrm{Day}<16.8\;\text{days},\mathrm{AGP}\left(t\right)=2.18-0.07\times \mathrm{Day}$ $\mathrm{Day}\geq 16.8\;\text{days},\mathrm{AGP}\left(t\right)=1$
Blood flow			
Cardiac index (measurement of cardiac output)	↑40%	[19]	$\mathrm{BF}\left(t\right)={\mathrm{BF}}_{1}\times \left(1.4-0.4\times \left(1-e^{-0.0274\times t}\right)\right)$
Gastric emptying time	↑45 min	—	Optimized

Abbreviations: BF, blood flow; GFR, glomerular filtration rate.

#### 2.4.1 Blood Flow

Longitudinal data for change of blood flow during malaria infection is scarce. Therefore, we employed an exponential interpolation approach to model the gradual normalization of this parameter. For example, the relative recovery of liver blood flow (LBF) at time *t* was described by Equation (1).

(1) $$\mathrm{LBF}\left(t\right)={\mathrm{LBF}}_{1}+\left({\mathrm{LBF}}_{2}-{\mathrm{LBF}}_{1}\right)\times \left(1-e^{-k\left(t-t_{1}\right)}\right)$$

where ${\mathrm{LBF}}_{1}$ represents relative LBF at the acute phase (time *t*_1_, 0 h) and ${\mathrm{LBF}}_{2}$ represents relative LBF at recovery (time *t*_2_, 168 h), assumed to be 1. *k* is the rate constant that determines the speed of recovery, which can be calculated by the known value *t*_2_ (Equation 2).

(2) $$k=-\frac{\log\left(0.01\right)}{t_{2}-t_{1}}$$

#### 2.4.2 Estimated Glomerular Filtration Rate (eGFR)

Dynamic eGFR data during malaria infection is absent; therefore, we used the same exponential interpolation to model this recovery process (Equations 1 and 2).

#### 2.4.3 Plasma Protein Level

AGP dynamics associated with malaria infection have been reported previously, which showed a linear reduction of AGP over time. Here, we adopt this reported equation directly to model the AGP recovery with an additional spline function to avoid a negative AGP value after Day 17.

There is no longitudinal data for albumin reported in the literature, so we used the same exponential interpolation to describe its dynamics, as described above (Equations 1 and 2).

#### 2.4.4 Metabolism

Dynamic change of metabolic enzymes during malaria infection is very limited, thus we applied an exponential equation to describe its dynamics (Table 1).

Here, we used a reported reduction value (%) of phase 1 enzyme protein levels (at Day 8 postinoculation representing parasitemia peak) as the maximum relative inhibition effect of enzyme associated malaria infection [15]. For UGT enzymes, mRNA levels were used instead of protein levels due to the lack of available protein data. Furthermore, assuming reversible inhibition of liver metabolic enzymes during malaria infection, we employed an exponential recovery equation to describe the time‐varying behavior of enzyme activity throughout the infection. For example, the CYP protein level (reference concentration in PK‐SIM) at time 𝑡 for a specific CYP enzyme isoform can be modeled using Equation (3).

(3) $$\mathrm{CYP}\left(t\right)={\mathrm{CYP}}_{1}+\left({\mathrm{CYP}}_{\text{normal}}-{\mathrm{CYP}}_{1}\right)\times \left(1-e^{-\frac{0.693\times t}{t_{1/2}}}\right)$$

where ${\mathrm{CYP}}_{1}$ and ${\mathrm{CYP}}_{\text{nomal}}$ represent the CYP protein levels during the acute phase of the disease (time *t*_1_, 0 h) and during the recovery stage of malaria (time *t*_2_), respectively. *t*_1/2_ is the human CYP enzyme turnover half‐life. ${\mathrm{CYP}}_{\text{normal}}$ value is available in the PK‐SIM library. The *t*_1/2_ values of 23, 23, 104, 26, 70, 27, 79, and 24 h for CYP2C8, CYP2B6, CYP2C9, CYP2C19, CYP2D6, CYP2E1, CYP3A4, and UGTs were used in Equation (3), respectively [22, 23].

#### 2.4.5 Absorption

During development of compound models in healthy volunteers, the default gastric parameters were used, and absorption lag time was optimized if necessary. During the verification of virtual malaria population, the gastric emptying time was further optimized to accurately capture absorption phase, as some clinical PK studies have suggested that this parameter could be increased during an acute malaria infection. This average value of optimized gastric emptying time was then implemented in the virtual malaria population.

### 2.5 Data Extraction

The relevant data for compound models and the virtual malaria population were extracted from the available literature. For studies where data were presented in graphical form (e.g., PK curves), Engauge Digitizer (version 4.1) was used to extract concentration‐time data. The extracted data were then compiled for subsequent analysis and comparison.

### 2.6 Implementation in MoBi

The virtual malaria population was developed using the MoBi platform, starting with an in‐built virtual healthy population in the PK‐SIM software. Modifications were made to specific biological parameters associated with uncomplicated malaria infection within the “Spatial Structures” module. These modifications included the parameters specified above, such as organ blood flow, metabolism, eGFR, plasma albumin and α1‐acid glycoprotein, and gastric parameters. These parameters were adapted using time‐varying equations to reflect the dynamic physiological changes that occur during the malaria infection, ensuring a more accurate representation of the affected population.

The reference concentration of a specific liver Phase 1 or Phase 2 enzyme was modified within the “Molecular Module” using an exponential recovery equation (as described earlier). In instances where in vitro enzyme kinetic parameters (from microsome or hepatocyte systems) were unavailable, in vivo clearance data were utilized through a retrograde model. The time‐varying in vivo clearance was then implemented in the “Reaction Module” to account for dynamic changes over time.

### 2.7 Simulation

The PBPK model was developed and evaluated using a healthy population and a developed virtual malaria population. In the simulation, the key attributes, including race, age, weight, and body mass index, were generated to align with the study population. The model predicted performance for both virtual populations was compared to assess whether the implementation of physiological changes associated with malaria infection led to improvements in prediction accuracy. A 30% coefficient of variation (CV) around the predicted median PK concentrations was added to reflect the expected PK variability.

### 2.8 Model Evaluation Criteria

Model predictive performance was evaluated using an average fold error (AFE) of simulated PK concentrations (Equation 4). AFE within a twofold error indicates adequate model performance. Prediction bias for PK exposure parameters (AUC and *C*_max_ by NCA approach [24]) was assessed using the corresponding ratios (Equations 5 and 6). Ratios within a twofold range were considered an acceptable prediction accuracy.

(4) $$\mathrm{AFE}=10^{\frac{1}{n}\sum \log\left(\frac{\text{Predicted}}{\text{Observed}}\right)}$$

(5) $$\mathrm{AUC}\;\text{ratio}=\frac{\text{Predicted}\;\mathrm{AUC}}{\text{Observed}\;\mathrm{AUC}}$$

(6) $$C_{\max}\;\text{ratio}=\frac{\text{Predicted}\;C_{\max}}{\text{Observed}\;C_{\max}}$$

### 2.9 Sensitivity Analysis

A local sensitivity analysis was conducted to assess how selected model parameters influenced the PK exposure (i.e., AUC and *C*_max_). The biological parameters associated with acute malaria infection, which were incorporated into the development of the virtual population, were selected for this analysis. There parameters were (1) liver enzyme abundance (reference concentration of phase 1 and phase 2 liver metabolic enzymes), (2) plasma AGP and albumin level, (3) organ blood flow, and (4) renal function (eGFR).

The impact of these parameters on the PK exposure was evaluated by altering the value of each parameter by ±20% [25]. The sensitivity coefficient (SC) was computed as seen in Equation (7) [25].

(7) $$\mathrm{SC}=\frac{\%∆Y}{\%∆P}$$

where *Y* is the value of the PK exposure; Δ*Y* is the percent change in the predicted PK exposure; *P* is the parameter value; and Δ*P* is the percent change in the model parameter value. If the calculated absolute SC value is higher than 0.5 (i.e., a 100% change of the assessed parameter leads to a ±50% alteration in PK exposure), it indicates that this parameter is very influential on the predicted PK exposure (high sensitivity). Parameters with an absolute SC value between 0.2 and 0.5 were considered as being moderately influential (moderate sensitivity), while absolute SC values of < 0.2 were not considered to be influential (low sensitivity) [26, 27].

The PK exposure (AUC and *C*_max_) during the first dosing interval for quinine, DHA, amodiaquine, and desethylamodiaquine were summarized for the sensitivity analysis, as this period reflects the most severe disease condition. Additionally, AUC over the entire observation period (i.e., Days 0–28) was also summarized for desethylamodiaquine, because of its long half‐life.

## 3 Results

### 3.1 Biological Parameters for Uncomplicated Malaria Infection

The major changes in biological parameters during acute malaria infection were summarized from existing literature. Quantitative values for these changes, along with the corresponding time‐varying equations describing their dynamics, were compiled and are presented in Table 1.

Briefly, at the acute malaria stage, liver CYP and UGT enzyme protein levels were significantly decreased by 25%–40%, with recovery dependent on their respective turnover half‐lives. eGFR and plasma albumin levels were only slightly affected, while plasma AGP levels showed a large increase during acute infection. Additionally, cardiac output was substantially elevated by 40%.

### 3.2 Observed PK Data

The basic characteristics of the PK studies included for PBPK model verification are summarized in Table S1. These studies encompass clinical PK data from both healthy volunteers (HVs) and patients with uncomplicated malaria for the selected anti‐malarial drugs.

PK studies in HVs were conducted following single‐dose administration, while patient studies were performed using clinical dose regimens. For quinine, two studies collected PK samples during both the acute and convalescence phases. Additionally, one study collected PK samples at each dosing occasion for DHA.

### 3.3 Compound Models

The compound‐specific parameters used for PBPK predictions are listed in Tables S2–S4. These parameters were employed to predict PK profiles in healthy subjects and verified against observed PK data. During the verification process, some parameters were optimized as needed.

#### 3.3.1 Quinine

In the quinine HVs PBPK model, the log*P* and *V*_max_ parameters were refined using observed IV PK data [28]. Additionally, parameters resulting in a Lag time for oral dosing were further optimized based on observed oral PK data [29, 30]. The model successfully predicted PK concentrations, with overall fold‐errors for *C*_max_ and AUC remaining below 1.5, demonstrating good agreement with observed data (Figure S1 and Table S5).

#### 3.3.2 Dihydroartemisinin

In the DHA compound model, Lag time for oral dosing was optimized based on observed oral PK data. Overall, the PBPK model prediction showed a good agreement with the observed PK data in HVs (Figure S2). The overall prediction bias for *C*_max_ and AUC were below 1.5‐fold error (Table S6).

#### 3.3.3 Amodiaquine/Desethylamodiaquine

For the amodiaquine model, log*P* and *k*_cat_ were optimized. Similarly, in the desethylamodiaquine model, log*P* and hepatic clearance (CL_H_) were modified to fit the observed data. As shown in Figure S3 and Table S7, the PBPK model prediction for amodiaquine and desethylamodiaquine in HVs population showed a good agreement with the observed PK data. Most of the observed PK data points fell within the model predictions, and the overall predicted PK exposure parameters (i.e., AUC and *C*_max_) were within a 1.5‐fold error of the observed values.

### 3.4 PBPK Prediction in Malaria Patients

#### 3.4.1 Quinine

**FIGURE 2:**
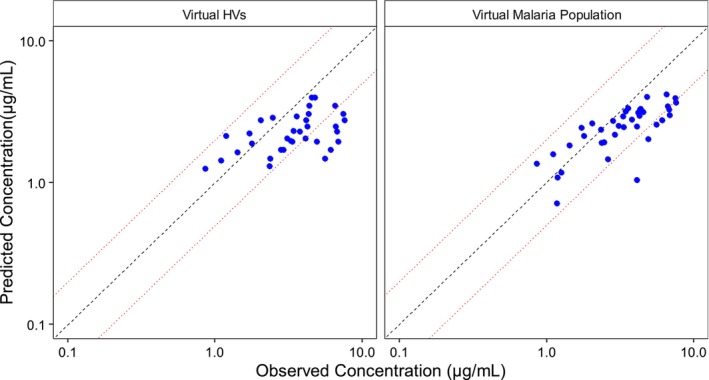
Correlation between observed quinine PK concentrations and the PBPK predictions when using a virtual healthy volunteer population (left panel) or the developed virtual malaria population (right panel).

The developed PBPK model, which integrated the quinine compound model with a virtual HVs population, under‐predicted quinine PK concentrations in malaria‐infected patients, particularly during the acute malaria phase. However, when the virtual malaria population was used for PBPK predictions, model performance assessed by AUC was improved by more than 29% for 2/4 studies, while predictions for 𝐶_max_ were similar across all four studies. The majority of fold‐errors for 𝐶_max_ and AUC fell below 1.5, as shown in Table 2, with detailed results provided in Table S5. The model predicted PK profiles are presented in Figure S4. As shown in Figure 2, the correlation between observed and predicted concentrations improved when using the virtual malaria population compared with the virtual healthy population.

**TABLE 2:** AUC and *C*_max_ ratios for PBPK predictions of quinine, dihydroartemisinin, amodiaquine, and desethylamodiaquine using a healthy volunteer (HV) population and the developed virtual malaria population.

Study	Virtual HV	Virtual malaria	Virtual HV	Virtual malaria
	*C*_max_ ratio	*C*_max_ ratio	AUC ratio	AUC ratio
Quinine				
Babalola 1998—acute [31]	1.42	1.26	1.71	1.14
Babalola 1998—convalescence [31]	0.76	0.76	0.79	1.30
Supanaranond 1991—acute [12]	1.88	1.91	2.81	2.00
Supanaranond 1991—convalescence [12]	1.13	1.27	1.62	1.43
Dihydroartemisinin				
Le Thi 2008 [13]	0.84	1.30	0.32	0.86
Nguyen 2009 [32]	0.60	0.76	0.35	0.79
Rijken 2011 [33]	1.01	1.07	0.41	1.09
Amodiaquine				
Scarsi 2014 [34]	1.88	1.41	0.96	0.92
TACT 2022 [8]	1.69	1.35	1.25	1.22
TRACTII 2020 [9]	1.73	1.33	1.28	1.24
Winstanley 1990 [35]	1.60	1.28	1.48	1.37
Desethylamodiaquine				
Scarsi 2014 [34]	0.73	0.62	0.65	0.62
TACT 2022 [8]	2.23	1.51	2.63	1.68
TRACTII 2020 [9]	2.39	1.56	2.20	1.38
Winstanley 1990 [35]	1.46	0.96	2.25	1.40

*Note:* The AUC and *C*_max_ ratios were calculated as the ratio of predicted values to observed values.

#### 3.4.2 Dihydroartemisinin

**FIGURE 3:**
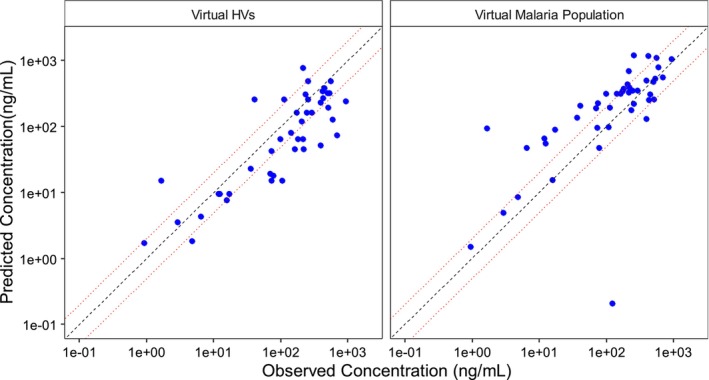
Correlation between observed dihydroartemisinin PK concentrations and the PBPK predictions when using a virtual healthy volunteer population (left panel) or the developed virtual malaria population (right panel).

The developed PBPK model, which integrated the dihydroartemisinin compound model with a virtual HVs population, under‐predicted dihydroartemisinin PK concentrations in malaria‐infected patients, particularly after the first dose. However, when the virtual malaria population was used for PBPK predictions, the model prediction assessed by AUC was improved by more than 2.2 folds for all three studies, while predictions for 𝐶_max_ were improved by more than 25% for 2/3 studies. The majority of fold‐errors for 𝐶_max_ and AUC fell below 1.5, as shown in Table 2, with detailed results provided in Table S6. Figure S5 depicts the model predicted PK. Figure 3 showed that the correlation between observed and predicted concentrations improved when using the virtual malaria population compared with the virtual healthy population.

#### 3.4.3 Amodiaquine and Desethylamodiaquine

The PBPK model with integration of the amodiaquine and desethylamodiaquine compound model with a virtual HVs population under‐predicted amodiaquine PK concentrations during the acute malaria infection. However, when the developed virtual malaria population was applied for PBPK predictions, the model prediction for amodiaquine assessed by *C*_max_ ratio improved by more than 20% for all studies, while AUC prediction ratios were similar for all four studies. The prediction of *C*_max_ ratios for desethylamodiaquine improved by more than 32% for 3/4 studies, while AUC predictions improved by more than 36% in 3 of 4 studies.

**FIGURE 4:**
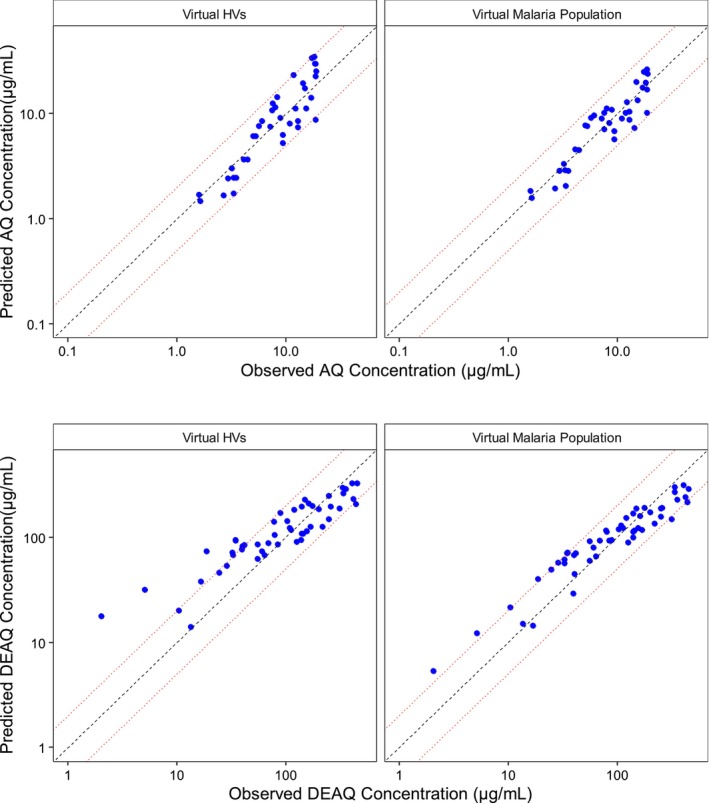
Correlation between observed amodiaquine and desethylamodiaquine PK concentrations and the PBPK predictions when using a virtual healthy volunteer population (left panel) or the developed virtual malaria population (right panel).

The majority of fold‐errors for 𝐶_max_ and AUC fell below 1.5, as shown in Table 2, with detailed results provided in Table S7. Figure S6 depicts the model predicted PK profiles. The correlation between observed and predicted concentrations improved when using the virtual malaria population relative to the virtual healthy population (Figure 4).

### 3.5 Sensitivity Analysis

**FIGURE 5:**
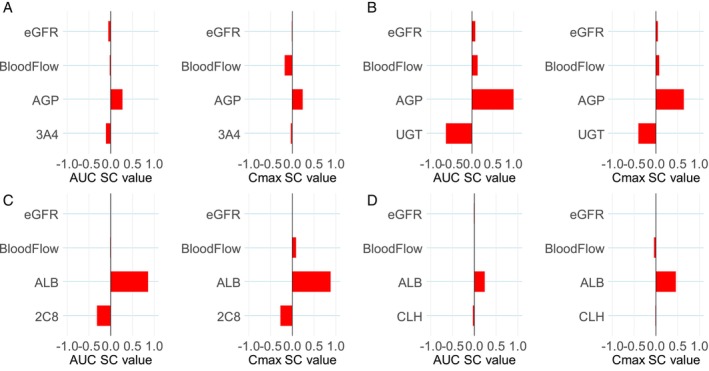
Sensitivity analysis of selected biological parameters associated with acute uncomplicated malaria infection on PK exposure of the first dose interval. (A) Quinine, (B) Dihydroartemisinin, (C) Amodiaquine, (D) Desethylamodiaquine. The sensitivity analysis was based on Babalola 1998 [31] (for quinine), Nguyen 2009 [32] (for dihydroartemisinin), and TACT‐CV 2022 [8] (for amodiaquine and desethylamodiaquine).

As shown in Figure 5, the selected biological parameters associated with a change during an acute uncomplicated malaria infection have various impacts on PK exposure. In general, AGP level has the largest sensitivity coefficient (SC) value on AUC and *C*_max_, indicating a substantial influential effect. Enzyme abundance has a moderate effect on PK exposures. Blood flow and eGFR have mild effects on PK exposure.

The impact of these biological changes on desethylamodiaquine AUC_0–28 day_ was minimal (SC < 0.03), with the exception of albumin. The effect of albumin was moderate, with an SC value of 0.239.

## 4 Discussion

In this study, we developed and evaluated a virtual population for uncomplicated malaria by incorporating significant biological changes into a PBPK modeling framework. Using this approach, the virtual population, combined with compound models, improved the prediction of PK profiles of several anti‐malarial drugs during acute uncomplicated malaria infection compared with using virtual healthy population, although the extent of improvement varied across compounds and studies. This advancement may help bridge the data gap in PK predictions for malaria target populations and provides a proof‐of‐concept translational framework to support the development of novel anti‐malarial therapies as well as dose optimization of currently available therapies.

The development of new anti‐malarial drugs remains an unmet medical need, particularly in the face of emerging and spreading resistance to existing treatments. PK plays a critical role in driving the efficacy and safety of candidate compounds during preclinical and clinical development. Accurate PK predictions in malaria‐infected patients are essential for optimizing clinical dosage regimens, ensuring that drug concentrations fall within the desired therapeutic range. This approach helps mitigate the risk of selecting a suboptimal dose, which could lead to therapeutic failure and development of drug resistance in a patient population. Traditionally, human PK predictions in drug discovery stage rely on allometric scaling and PBPK modeling approaches, with the latter gaining increasing popularity in recent years. Accordingly, PBPK predictions can be further refined using real phase 1 PK data from healthy volunteers. Phase 2 dose selection often assumes minimal PK differences between healthy volunteers and patient populations. This assumption holds only when disease‐related biological changes do not significantly impact a drug's absorption, distribution, metabolism, and/or excretion (ADME) properties. Emerging evidence suggests that this assumption is not valid in malaria‐infected patients, as PK properties differ substantially for some anti‐malaria drugs [12, 13, 31]. Thus, PK predictions based solely on a virtual healthy population have limited extrapolative ability for malaria patients. It is essential to develop a virtual malaria‐infected population within a PBPK modeling framework to improve the accuracy of PK predictions and optimize dosing strategies in clinical development.

Biological changes associated with uncomplicated malaria infection can be large, such as a substantially increased (~40%) cardiac output [19]. This elevation leads to increased liver blood flow (*Q*_H_), which, under the well‐stirred model assumption, enhances the liver's maximum metabolic capacity of high‐extraction drugs. Another small clinical study reported a 55% increase in liver blood flow during the acute phase of uncomplicated malaria, as determined by the plasma clearance of Indocyanine Green [36]. This increase was consistent with the observed rise in cardiac output. In this analysis, we reasonably assumed that organ blood flow, including liver blood flow, increases proportionally to cardiac output. Data on human liver enzyme changes during malaria infection are scarce. The available animal studies suggested that malaria infection significantly impacts liver enzyme activity. In mouse models, Phase 1 and Phase 2 enzyme activities were reported to be substantially decreased during malaria infection, with the lowest protein levels observed at the peak of parasitemia [15, 37, 38]. Although this data was obtained from preclinical experiments, the model prediction improved when implementing this enzyme reduction in the developed PBPK framework for virtual malaria patients.

Plasma proteins, such as albumin and AGP, play a crucial role in the PK of anti‐malarial drugs. These proteins show varying changes during malaria infection. In the acute phase, AGP levels increase approximately twofold, with the potential of significantly impacting the PK of drugs, particularly strongly bound basic drugs as many of the anti‐malarial partner drugs. In contrast, albumin levels show a slight decrease, which is expected to have a minimal effect on the PK of bound drugs [17]. In this study, we assumed that the recovery time for albumin was the same as that of AGP, with both returning to baseline levels approximately 17 days after anti‐malarial treatment [18].

In the development and evaluation of the virtual malaria population, we selected three anti‐malarial drugs—quinine, dihydroartemisinin, and amodiaquine—covering a range of metabolic pathways (CYP3A4, CYP2C8, and UGT2B7), protein binding characteristics (albumin and AGP), and elimination half‐lives (from a few hours to as long as 10 days). This selection ensures broader applicability and confidence in extrapolating the model to other anti‐malarial drugs. We observed that PBPK predictions using a virtual healthy population substantially under‐predicted the PK profiles of dihydroartemisinin, quinine, and desethylamodiaquine during the acute phase of malaria. This under‐prediction suggests that patients with uncomplicated malaria infection may be exposed to higher drug concentrations than anticipated, potentially increasing safety risks. In contrast, the PBPK model for amodiaquine using a virtual healthy population overpredicted the PK, most likely due to its high hepatic extraction rate, where liver blood flow plays a dominant role in drug metabolism. Overall, PBPK predictions improved when using a virtual malaria population, providing potential utility and a proof‐of‐concept translational framework to capture disease‐specific physiological changes. This virtual malaria population, implemented in MoBi, can be used to accurately predict the PK of new candidate compounds in acute uncomplicated malaria infection.

Moreover, we quantitatively evaluated the impact of these biological changes on PK exposure using sensitivity analysis. AGP was identified as the biological parameter exerting the greatest influence on the PK exposure of anti‐malarial drugs that predominantly bind to this plasma protein. In contrast, plasma albumin also showed a notable effect on the PK exposure of drugs with predominant albumin binding. However, the average reduction in albumin (−16%) during the acute phase was an order of magnitude lower compared with the increase in AGP. Thus, a 16% decrease in albumin is unlikely to result in a substantial reduction in PK exposure. A reduced enzyme abundance associated with acute malaria infection was also evident in a murine malaria model [15, 37], and this finding was corroborated in the current PBPK model through a sensitivity analysis, which showed a significant impact on PK exposure. In the context of elevated AGP levels, an additional reduction in enzyme abundance could further increase PK exposure. Interestingly, organ blood flow exhibited differential effects on the anti‐malarial drugs studied. For quinine and desethylamodiaquine, an increase in blood flow led to a reduction in PK exposure. This can be explained by the fact that both drugs have a large volume of distribution [20, 39, 40, 41, 42] and an intermediate hepatic extraction ratio and thus higher blood flow enhances tissue distribution, thereby lowering systemic exposure. In contrast, the PK exposure of dihydroartemisinin and amodiaquine slightly increased with elevated blood flow. This occurs because increased hepatic blood flow reduces the extent of first‐pass metabolism (reduce extraction ratio), resulting in less drug being metabolized during the first pass and consequently higher bioavailability.

The most clinically used anti‐malarial drugs are predominantly eliminated by hepatic pathways, and the compounds selected in this study were intended to reflect this therapeutic class. Among anti‐malarial agents, chloroquine exhibits a substantial contribution of renal excretion, accounting for approximately half of total clearance after a single dose [43, 44]. However, chloroquine also undergoes significant hepatic metabolism (e.g., via CYP3A4 and CYP2C8) [45], and therefore does not represent a typical renally eliminated compound. Hydroxychloroquine has an even lower contribution of renal excretion (approximately 16%–30% of total clearance) [46], and these two compounds were therefore not considered suitable as model drugs for major renal elimination. In addition, a mixed‐elimination compound (quinine) with 20% renal clearance has been included and evaluated in the current framework. Therefore, a model drug with predominantly renal elimination was not included in the present work.

Severe forms of malaria infection, such as cerebral malaria, have significantly reduced blood flow kinetics and impaired renal function compared to uncomplicated malaria infection [19, 47]. Moreover, malaria infection in specific populations, such as pregnant women, involves more complex physiological changes beyond those caused by the infection itself. Expanding the virtual malaria population to capture these conditions requires further data collection and targeted research to address existing knowledge gaps.

Our study has several key limitations. (1) The accuracy of the overall predictions varied across studies and drugs, and further studies are required to refine the virtual malaria population and improve predictions. (2) The changes in plasma albumin and AGP levels exhibit distinct patterns during the inflammatory response associated with acute malaria infection. In the present analysis, we accounted only for the primary binding plasma protein. The interplay between these two major plasma proteins requires further refinement in future work. (3) The liver enzyme activity reduction data during acute malaria infection was generated from a murine malaria model, which may affect the results. (4) Due to the lack of data, the recovery time for organ blood flow was assumed to be 7 days after the treatment; this needs to be further refined. (5) We assumed 30% CV for median PK prediction as variability. Population predictions, including variability estimates of key input parameters, might more accurately describe the variability of the target population. This needs to be improved in future studies.

In conclusion, we developed and validated a virtual population representing an acute uncomplicated malaria infection. This virtual malaria population improved the prediction of anti‐malarial drug PK during the acute phase of infection and may serve as a useful translational framework to support the development of novel anti‐malarial therapies.

## Author Contributions

J.D., Q.P., and R.M.H. wrote the manuscript; J.D. and J.T. designed the research; J.D. and Q.P. performed the research; J.D. analyzed the data; J.D. and Q.P. contributed new reagents/analytical tools.

## Funding

This research was funded in whole or in part by the Wellcome Trust grant 220211.

## Conflicts of Interest

The authors declare no conflicts of interest.

## Supporting information


**Figure S1:** PBPK prediction of quinine in a healthy population.
**Figure S2:** PBPK prediction of dihydroartemisinin in a healthy population.
**Figure S3:** PBPK prediction of amodiaquine (a) and desethylamodiaquine (b) in a healthy population.
**Figure S4:** PBPK prediction of quinine using a healthy volunteer population (left panel) and a virtual malaria population (right panel).
**Figure S5:** PBPK prediction of dihydroartemisinin using virtual healthy (left panel) and malaria populations (right panel).
**Figure S6:** PBPK prediction of amodiaquine and desethylamodiaquine using virtual healthy (left panel) and malaria populations (right panel).
**Table S1:** PK studies for PBPK model verification.
**Table S2:** PBPK input parameters for quinine.
**Table S3:** PBPK input parameters for dihydroartemisinin.
**Table S4:** PBPK input parameters for amodiaquine and desethylamodiaquine.
**Table S5:** AUC and *C*_max_ ratios for PBPK predictions of quinine using a healthy volunteer population and the developed virtual malaria population.
**Table S6:** AUC and *C*_max_ ratios for PBPK predictions of dihydroartemisinin using a healthy volunteer population and the developed virtual malaria population.
**Table S7:** AUC and *C*_max_ ratios for PBPK predictions of amodiaquine and desethylamodiaquine using a healthy volunteer population and the developed virtual malaria population.

**Data S1:** Quinine MoBi model—virtual malaria population.
**Data S2:** Dihydroartemisinin MoBi model—virtual malaria population.
**Data S3:** Amodiaquine and desethylamodiaquine MoBi model—virtual malaria population.

## Data Availability

The data supporting the findings of this study are available from the corresponding author upon reasonable request.
