# Supplementary material for: Development of a Disease‐Specific Virtual Malaria Population for Physiologically‐Based Pharmacokinetic Modeling
Source: CPT Pharmacometrics Syst Pharmacol. 2026 Jul 16;15(8):e70294. doi: 10.1002/psp4.70294 (PMC13375944; doi:10.1002/psp4.70294)
Supplement: Supplementary file 1 — Figure S1: PBPK prediction of quinine in a healthy population. Figure S2: PBPK prediction of dihydroartemisinin in a healthy population. Figure S3: PBPK prediction of amodiaquine (a) and desethylamodiaquine (b) in a healthy population. Figure S4: PBPK prediction of quinine using a healthy volunteer population (left panel) and a virtual malaria population (right panel). Figure S5: PBPK prediction of dihydroartemisinin using virtual healthy (left panel) and malaria populations (right panel). Figure S6: PBPK prediction of amodiaquine and desethylamodiaquine using virtual healthy (left panel) and malaria populations (right panel). Table S1: PK studies for PBPK model verification. Table S2: PBPK input parameters for quinine. Table S3: PBPK input parameters for dihydroartemisinin. Table S4: PBPK input parameters for amodiaquine and desethylamodiaquine. Table S5: AUC and C max ratios for PBPK predictions of quinine using a healthy volunteer population and the developed virtual malaria population. Table S6: AUC and C max ratios for PBPK predictions of dihydroartemisinin using a healthy volunteer population and the developed virtual malaria population. Table S7: AUC and C max ratios for PBPK predictions of amodiaquine and desethylamodiaquine using a healthy volunteer population and the developed virtual malaria population. [file PSP4-15-e70294-s002.docx]

**SUPPLEMENTARY MATERIAL**

**Development of a Disease-Specific Virtual Malaria Population for Physiologically-based Pharmacokinetic Modeling**

*J. Ding, Q. Pei, R.M. Hoglund, J. Tarning*

**Supplementary Tables and Figures**

Table S1. PK studies for PBPK model verification.

Table S2. PBPK input parameters for quinine.

Table S3. PBPK input parameters for dihydroartemisinin.

Table S4. PBPK input parameters for amodiaquine and desethylamodiaquine.

Table S5. AUC and Cmax ratios for PBPK predictions of quinine using a healthy volunteer population and the developed virtual malaria population.

Table S6. AUC and Cmax ratios for PBPK predictions of dihydroartemisinin using a healthy volunteer population and the developed virtual malaria population.

Table S7. AUC and Cmax ratios for PBPK predictions of amodiaquine and desethyl-amodiaquine using a healthy volunteer population and the developed virtual malaria population.

Figure S1. PBPK prediction of quinine in a healthy population.

Figure S2. PBPK prediction of dihydroartemisinin in a healthy population.

Figure S3. PBPK prediction of amodiaquine (a) and desethylamodiaquine (b) in a healthy population.

Figure S4. PBPK prediction of quinine using a healthy volunteer population (left panel) and a virtual malaria population (right panel).

Figure S5. PBPK prediction of dihydroartemisinin using virtual healthy (left panel) and malaria populations (right panel).

Figure S6. PBPK prediction of amodiaquine and desethyl-amodiaquine using virtual healthy (left panel) and malaria populations (right panel).

**Supplementary References**

**Table S1. PK studies for PBPK model verification.**

| **Authors/year** | **Population** | **Age**  **range (years)** | **Weight range (kg)** | **Number of**  **subjects** | **Dosage regimen** |
| --- | --- | --- | --- | --- | --- |
| **Amodiaquine/desethylamodiaquine** | | | | | |
| Winstanley 1987(1) | HVs | 22-46 | NA | 18 | A single 200, 400, 600 mg oral dose |
| Winstanley 1987(2) | HVs | 22-44 | NA | 7 | A single 600 mg oral dose |
| Pussard 1987(3) | HVs | 27-50 | 46-80 | 4 | A single 10 mg/kg oral dose |
| Orrell 2008(4) | HVs | Average 24.4 | Average 67.3 | 15 | 10 mg/kg alone or plus artesunate, single dose |
| Winstanley 1990(5) | Patients | 15-55 | 44-78 | 14 | 10 mg/kg, followed by 5 mg/kg at 6 h, 24 h and 48 h |
| Scarsi 2014 (6) | Patients | Average 35.8 | NA | 11 | 600 mg of AQ + artesunate oral for 3 days |
| TACT-CV 2022(7)  TACTII 2020(8) | Patients | 8.0-58.4 | 18-78 | 99 | 225 mg of AQ for 6 doses with AL |
| **Dihydroartemisinin** | | | | | |
| Hong 2008(9) | HVs | Average 27 | Average 60 | 18 | A single oral dose of 80 mg DHA |
| Chinh 2009(10) | HVs | Average 21 | Average 60 | 24 | A single oral dose of 120 mg DHA/960 mg of piperaquine (PQ) |
| Hanboonkunupakarn 2014(11) | HVs | 24-52 | 54-71.4 | 16 | A single oral dose of 120 mg DHA/960 mg of PQ |
| Reuter 2015(12) | HVs | Average 24.4 | Average 81.6 | 19 | A single oral dose of 160 mg DHA/1280 mg of PQ |
| Le Thi 2008(13) | Patients | 17-47 | 46-64 | 10 | 300, 300, 100, 100 and 100 mg daily oral dose |
| Nguyen 2009(14) | Patients | Average 26.3 | Average 56.5 | 12 | 3-day course of DHA-OQ (2.4 mg/kg DHA and 19.2 mg/kg of PQ daily) |
| Rijken 2011(15) | Patients | Average 30.5 | Average 47 | 24 | 3-day course of DHA-PQ (a total dose of 6.4 mg/kg DHA and 51.2 mg/kg of piperaquine) |
| **Quinine** | | | | | |
| Karbwang 1993(16) | HVs | 19-27 | NA | 8 | A single dose of 10 mg/kg quinine base IV infusion over 1 hours |
| Babalola 1997(17) | HVs | 19-28 | 50-68 | 7 | A single oral dose of 1000 mg quinine base |
| Igbinoba 2015(18) | HVs | 21-28 | 55-78 | 24 | A single oral dose of 500 mg quinine base |
| Supanaranond 1991(19) | Patients | 17-72 | 42-60 | 15 | A 7-day course of quinine base (8.6 mg/kg daily) at acute phase, and a single dose at convalescence period |
| Babalola 1998(20) | Patients | 8-20 | 18-55 | 6 | A 7-day course of quinine base (8.6 mg/kg daily) at acute phase, and a single dose at convalescence period |

**Table S2. PBPK input parameters for quinine.**

| **Parameter** | **Value** | **Source** |
| --- | --- | --- |
| **Physicochemical parameters** | | |
| Molecular weight (g/mol) | 324.42 | Drug bank |
| pKa | 8.5 (B), 4.2 (B) | (21) |
| Solubility (PBS7.4) | 806 ng/mL | (21) |
| Solubility (FaSSGF, pH=1.6) | 1925 ng/mL | (21) |
| Solubility (FaSSIF, pH=6.5) | 1960 ng/mL | (21) |
| Permeability | 39×10^-6^ cm/s | (22) |
| clogP | 2.25 | Parameter identification |
| LogD7.4 | 1.80 | (21) |
| **Distribution** |  |  |
| Partition coefficient | Poulin and Theil |  |
| Cellular permeability | PK-SIM standard |  |
| Fraction unbound (AGP) | 0.37 | (21, 23) |
| B:P ratio | 0.67 | (21) |
| **Metabolism and elimination** | | |
| **CYP3A4** | Vmax = 0.48 nM/min/nM  Km = 11.98 µM | Parameter identification  (24) |
| Biliary clearance | Negligible |  |
| Renal clearance | 20% | (25) |
| **Absorption (Weibull model)** |  |  |
| Dissolution time (50% dissolved) | 15 min | PK-SIM default value |
| Dissolution shape | 0.92 | PK-SIM default value |
| Gastric emptying time | 15 min (HVs),  60 min (Malaria) | PK-SIM default value  Optimized |
| Lag time | 33.2 min (HVs) | Optimized |

**Table S3. PBPK input parameters for dihydroartemisinin.**

| **Parameters** | **Value** | **Source** |
| --- | --- | --- |
| **Physicochemical parameters** | | |
| Molecular weight (g/mol) | 284.35 | Drug bank |
| pKa | n/a (Neutral) |  |
| Solubility (PBS7.4) | 130 µg/mL | (21) |
| Solubility (FaSSGF, pH=1.6) | 140 µg/mL | (21) |
| Solubility (FaSSIF, pH=6.5) | 159 µg/mL | (21) |
| Permeability | 5.0×10^-5^ cm/s | Caco2 cell (21) |
| clogP | 2.84 | (21) |
| LogD7.4 | 2.3 | (21) |
| **Distribution** |  |  |
| Partition coefficient | Rodgers & Rowland |  |
| Cellular permeability | PK-SIM standard |  |
| Fraction unbound (AGP) | 0.21 | (26) |
| B:P ratio | 1 | Not determine, unstable in blood and plasma |
| **Metabolism and elimination** | | |
| UGT1A9/UGT2BT | Clint = 145 µL/min/mg | Calculated from CL PO (273.2 L/h(27)) using the Retrograde model |
| Biliary clearance | Negligible |  |
| Renal clearance | Negligible | EMA report |
| **Absorption (Weibull model)** |  |  |
| Dissolution time (50% dissolved) | 15 min | PK-SIM default value |
| Dissolution shape | 0.92 | PK-SIM default value |
| Gastric emptying time | 15 min (HVs),  60 min (Malaria) | PK-SIM default value  Optimized |
| Lag time | 15 min (HVs, Malaria) | Optimized |

**Table S4. PBPK input parameters for amodiaquine and desethylamodiaquine.**

| **Parameters** | **Value** | **Source** | |  |
| --- | --- | --- | --- | --- |
| **Amodiaquine** | | | |  |
| **Physicochemical parameters** | | |  |  |
| Molecular weight (g/mol) | 355.86 | Drug bank | |  |
| pKa | 7.59 (B), 6.25 (B), 10.3 (A) | (21) | |  |
| Solubility (PBS7.4) | 26.3 µg/mL | (21) | |  |
| Solubility (FaSSGF, pH=1.6) | > 2000 µg/mL | (21) | |  |
| Solubility (FaSSIF, pH=6.5) | 1120 µg/mL | (21) | |  |
| Permeability | 1.6×10^-5^ cm/s | Caco2 cell (28) | |  |
| clogP | 3.51 | Calculated from LogD (2.95) | |  |
| **Distribution** |  |  | |  |
| Partition coefficient | PK-SIM standard |  | |  |
| Cellular permeability | PK-SIM standard |  | |  |
| Protein binding (Albumin) | 0.91 | (21) | |  |
| B:P ratio | 1.1 | (21) | |  |
| **Metabolism and elimination** | | | |  |
| rhCYP2C8 Yeast | Vmax = 3.9 pmol/min/pmol  Km = 0.9 µmol, Clint = 4.4 mL/min/pmol | (29) | |  |
| CYP2D6 and CYP3A4 | Relative contribution: 28%, (20.7×0.28=5.8 mL/min/mg ) | (30) | |  |
| Biliary clearance | Negligible | (31) | |  |
| Renal clearance | Negligible | (2) | |  |
| **Absorption model** | | | |  |
| Dissolution time (50% dissolved) | 3.7 min | (32) | |  |
| Dissolution shape | 0.92 | PK-SIM default value | |  |
| Gastric emptying time | 15 min (HVs),  60 min (Malaria) | PK-SIM default value  Optimized | | |
| Lag time | 0 min (HVs, Malaria) | PK-SIM default value | | |
| **Desethylamodiaquine** | | | | |
| **Physicochemical parameters** | | | | |
| Molecular weight (g/mol) | 327.8 | Drug bank | | |
| pKa | 8.0 (B), 7.1 (B) | (21) | | |
| Solubility (pH=7） | 7.76 µg/mL | ALOGPS | | |
| logP | 2.17 | Calculated from LogD (1.3) | | |
| logD_7.4_ | 1.30 | (21) | | |
| **Distribution** |  |  | | |
| Partition coefficient | PK-SIM standard |  | | |
| Cellular permeability | PK-SIM standard |  | | |
| Protein binding | 0.83 (AGP and albumin) | (33) | | |
| B:P ratio | 3.26 | (21) | | |
| **Metabolism and elimination** | | | | |
| Intrinsic clearance  (CYP2D6, 3A4, 2C8, 2C9) | Vmax = 5.5 pmol/min/mg  Km = 6.1 µmol/L  Clint = 0.9 µL/min/mg | (34) | | |

**Table S5. AUC and Cmax ratios for PBPK predictions of quinine using a healthy volunteer population and the developed virtual malaria population.**

| Study | Cmax_obs_ | HV Cmax_pred_ | HV Cmax  ratio | Mal Cmax_pred_ | Mal Cmax  ratio | AUC_obs_ | HV AUC_Pred_ | HV  AUC ratio | Mal AUC_Pred_ | Mal  AUC ratio | HV AFE | Mal AFE |
| --- | --- | --- | --- | --- | --- | --- | --- | --- | --- | --- | --- | --- |
| ***Healthy population*** |  |  |  |  |  |  |  |  |  |  |  |  |
| Babalola 1997 | 5.1 | 6.2 | 0.82 | - | - | 93.6 | 109.8 | 0.85 | - | - | 0.31 | - |
| Igbinoba 2015 | 3.8 | 4.4 | 0.87 | - | - | 56.5 | 54.9 | 1.03 | - | - | 1.00 | - |
| Karbwang 1993 | 4.1 | 5.8 | 0.70 | - | - | 42.6 | 36.2 | 1.18 | - | - | 0.89 | - |
| ***Malaria patients*** |  |  |  |  |  |  |  |  |  |  |  |  |
| Babalola 1998-Acute | 4.9 | 3.5 | 1.42 | 3.9 | 1.26 | 38.6 | 22.5 | 1.71 | 33.9 | 1.14 | 0.27 | 0.85 |
| Babalola 1998-Convalescence | 2.5 | 3.2 | 0.76 | 3.2 | 0.76 | 18.2 | 23.0 | 0.79 | 23.0 | 1.30 | 1.30 | 1.30 |
| Supanaranond 1991-Acute | 7.6 | 4.05 | 1.88 | 3.97 | 1.91 | 73.3 | 26.1 | 2.81 | 36.7 | 2.00 | 0.28 | 0.57 |
| Supanaranond 1991-Convalescence | 4.6 | 4.0 | 1.13 | 3.6 | 1.27 | 42.1 | 26.1 | 1.62 | 29.4 | 1.43 | 0.29 | 0.62 |

Cmax_obs_ and AUC_obs_ are the observed maximum concentration (Cmax) and area under the curve (AUC), from published data. Cmax_pred_ and AUC_pred_ are PBPK model predicted values using the virtual healthy population (HV) or the virtual malaria population (Mal). The AUC and Cmax ratios were calculated as the ratio of predicted values to observed values. AFE is the average fold error.

**Table S6. AUC and Cmax ratios for PBPK predictions of dihydroartemisinin using a healthy volunteer population and the developed virtual malaria population.**

| Study | Cmax_obs_ | HV Cmax_pred_ | HV Cmax  ratio | Mal Cmax_pred_ | Mal Cmax  ratio | AUC_obs_ | HV AUC_Pred_ | HV  AUC ratio | Mal AUC_Pred_ | Mal  AUC ratio | HV AFE | Mal AFE |
| --- | --- | --- | --- | --- | --- | --- | --- | --- | --- | --- | --- | --- |
| ***Healthy population*** |  |  |  |  |  |  |  |  |  |  |  |  |
| Chinh 2009 | 157 | 301 | 1.92 | - | - | 412 | 651 | 0.63 | - | - | 1.58 | - |
| Hanboonkunupakarn 2014 | 319 | 301 | 0.94 | - | - | 935 | 651 | 1.44 | - | - | 0.70 | - |
| Hong 2008 female | 212 | 148 | 0.70 | - | - | 530 | 469 | 1.13 | - | - | 0.89 | - |
| Hong 2008 male | 212 | 118 | 0.56 | - | - | 483 | 373 | 1.30 | - | - | 0.77 | - |
| Reuter 2015 | 224 | 312 | 1.39 | - | - | 649 | 714 | 0.91 | - | - | 1.10 | - |
| ***Malaria patients*** |  |  |  |  |  |  |  |  |  |  |  |  |
| Le Thi 2008 | 932 | 783 | 0.84 | 1213 | 1.30 | 15757 | 5059 | 0.32 | 13591 | 0.86 | 0.60 | 2.05 |
| Nguyen 2009 | 509 | 306 | 0.60 | 387 | 0.76 | 1963 | 678 | 0.35 | 1547 | 0.79 | 0.65 | 1.77 |
| Rijken 2011 | 535 | 543 | 1.01 | 573 | 1.07 | 6028 | 2473 | 0.41 | 6598 | 1.09 | 0.23 | 1.44 |

Cmax_obs_ and AUC_obs_ are the observed maximum concentration (Cmax) and area under the curve (AUC), from published data. Cmax_pred_ and AUC_pred_ are PBPK model predicted values using the virtual healthy population (HV) or the virtual malaria population (Mal). The AUC and Cmax ratios were calculated as the ratio of predicted values to observed values. AFE is the average fold error.

**Table S7. AUC and Cmax ratios for PBPK predictions of amodiaquine and desethyl-amodiaquine using a healthy volunteer population and the developed virtual malaria population.**

| Study | Cmax_obs_ | HV Cmax_pred_ | | HV Cmax  ratio | Mal Cmax_pred_ | Mal Cmax  ratio | AUC_obs_ | HV AUC_Pred_ | HV  AUC ratio | Mal AUC_Pred_ | Mal  AUC ratio | HV AFE | Mal AFE | |
| --- | --- | --- | --- | --- | --- | --- | --- | --- | --- | --- | --- | --- | --- | --- |
| **Amodiaquine** |  |  |  | |  |  |  |  |  |  |  |  | |  |
| ***Healthy population*** |  |  |  | |  |  |  |  |  |  |  |  | |  |
| Orrell 2008- 600 mg | 22.6 | 31.1 | 1.37 | | - | - | 196 | 151 | 0.77 | - | - | 0.88 | | - |
| Winstanley 1987- 600 mg | 31.0 | 28.5 | 0.92 | | - | - | 123 | 126 | 1.03 | - | - | 0.97 | | - |
| Winstanley 1987- 200 mg | 14.5 | 9.5 | 0.65 | | - | - | 25.7 | 29.9 | 1.16 | - | - | 0.99 | | - |
| Winstanley 1987- 400 mg | 17.0 | 19.0 | 1.11 | | - | - | 62.9 | 74.0 | 1.18 | - | - | 1.05 | | - |
| Winstanley 1987- 600 mg | 23.5 | 28.5 | 1.21 | | - | - | 106 | 126 | 1.19 | - | - | 1.12 | | - |
| ***Malaria patients*** |  |  |  | |  |  |  |  |  |  |  |  | |  |
| Scarsi 2014 | 18.7 | 35.2 | 1.88 | | 26.1 | 1.41 | 230 | 212 | 0.96 | 215 | 0.92 | 1.09 | | 0.99 |
| TACT 2022 | 7.5 | 12.7 | 1.69 | | 10.2 | 1.35 | 40.9 | 51.1 | 1.25 | 49.9 | 1.22 | 1.01 | | 1.06 |
| TRACTII 2020 | 8.4 | 14.5 | 1.73 | | 11.2 | 1.33 | 42.4 | 54.3 | 1.28 | 52.6 | 1.24 | 1.09 | | 1.16 |
| Winstanley 1990 | 18.9 | 30.3 | 1.60 | | 24.2 | 1.28 | 96.4 | 143 | 1.48 | 132 | 1.37 | 0.88 | | 0.84 |
| **desethyl-amodiaquine** |  |  |  | |  |  |  |  |  |  |  |  | |  |
| ***Healthy population*** |  |  |  | |  |  |  |  |  |  |  |  | |  |
| Orrell 2008- 600 mg | 194 | 178 | 0.92 | | - | - | 9601 | 12779 | 1.33 | - | - | 0.66 | | - |
| Winstanley 1987- 600 mg | 165 | 193 | 1.17 | | - | - | 5346 | 5252 | 0.98 | - | - | 0.84 | | - |
| Winstanley 1987- 200 mg | 38.0 | 64.5 | 1.70 | | - | - | 1911 | 1753 | 0.92 | - | - | 1.42 | | - |
| Winstanley 1987- 400 mg | 116 | 129 | 1.11 | | - | - | 4569 | 3504 | 0.77 | - | - | 1.12 | | - |
| Winstanley 1987- 600 mg | 192 | 193 | 1.00 | | - | - | 6034 | 5252 | 0.87 | - | - | 0.83 | | - |
| ***Malaria patients*** |  |  |  | |  |  |  |  |  |  |  |  | |  |
| Scarsi 2014 | 453 | 330 | 0.73 | | 280.2 | 0.62 | 21404 | 13963 | 0.65 | 13165 | 0.62 | 0.72 | | 0.60 |
| TACT 2022 | 41.7 | 93.0 | 2.23 | | 63.0 | 1.51 | 218 | 574 | 2.63 | 367 | 1.68 | 2.17 | | 1.42 |
| TRACTII 2020 | 40.1 | 95.9 | 2.39 | | 62.6 | 1.56 | 267 | 587 | 2.20 | 368 | 1.38 | 1.72 | | 1.15 |
| Winstanley 1990 | 162 | 238 | 1.46 | | 155.5 | 0.96 | 608 | 1366 | 2.25 | 850 | 1.40 | 1.67 | | 0.97 |

Cmax_obs_ and AUC_obs_ are the observed maximum concentration (Cmax) and area under the curve (AUC), from published data. Cmax_pred_ and AUC_pred_ are PBPK model predicted values using the virtual healthy population (HV) or the virtual malaria population (Mal). The AUC and Cmax ratios were calculated as the ratio of predicted values to observed values. AFE is the average fold error.


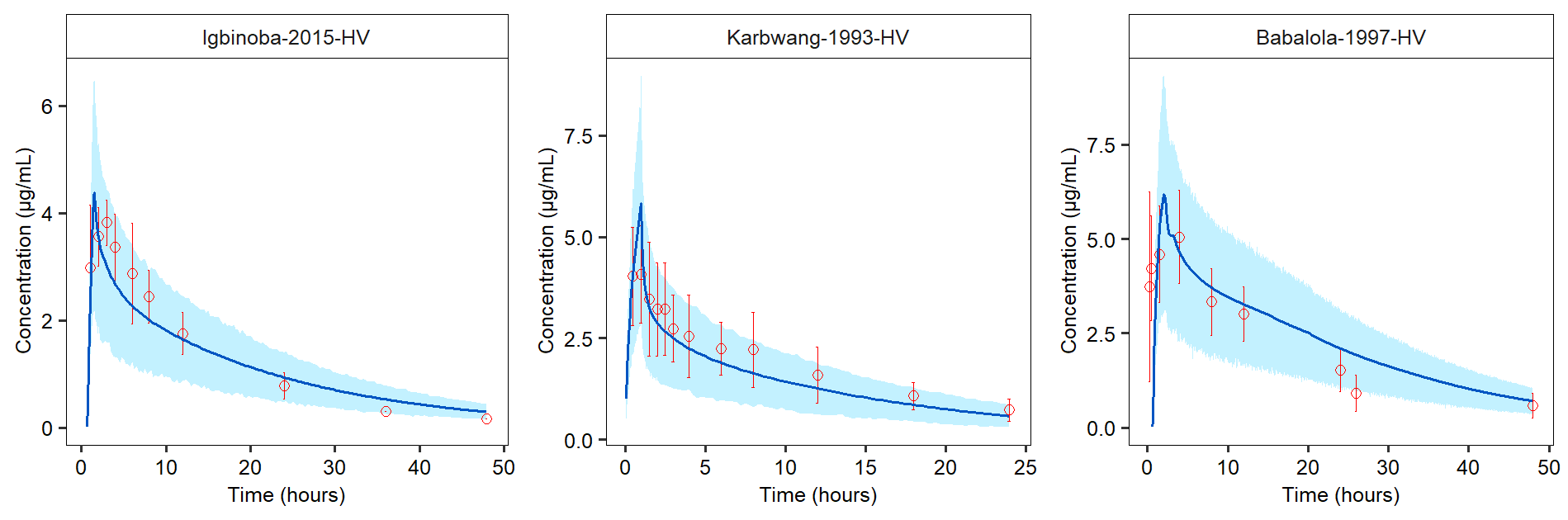


**Figure S1. PBPK prediction of quinine in a healthy population.**

The PBPK model incorporated key demographic elements (e.g., age, and weight) aligned with the published studies. The blue line represents the predicted median concentration, while the shaded region depicts a 30% coefficient of variation (CV) around the predicted median. Red dots and error bars indicate observed average PK concentrations and their standard deviations.

**
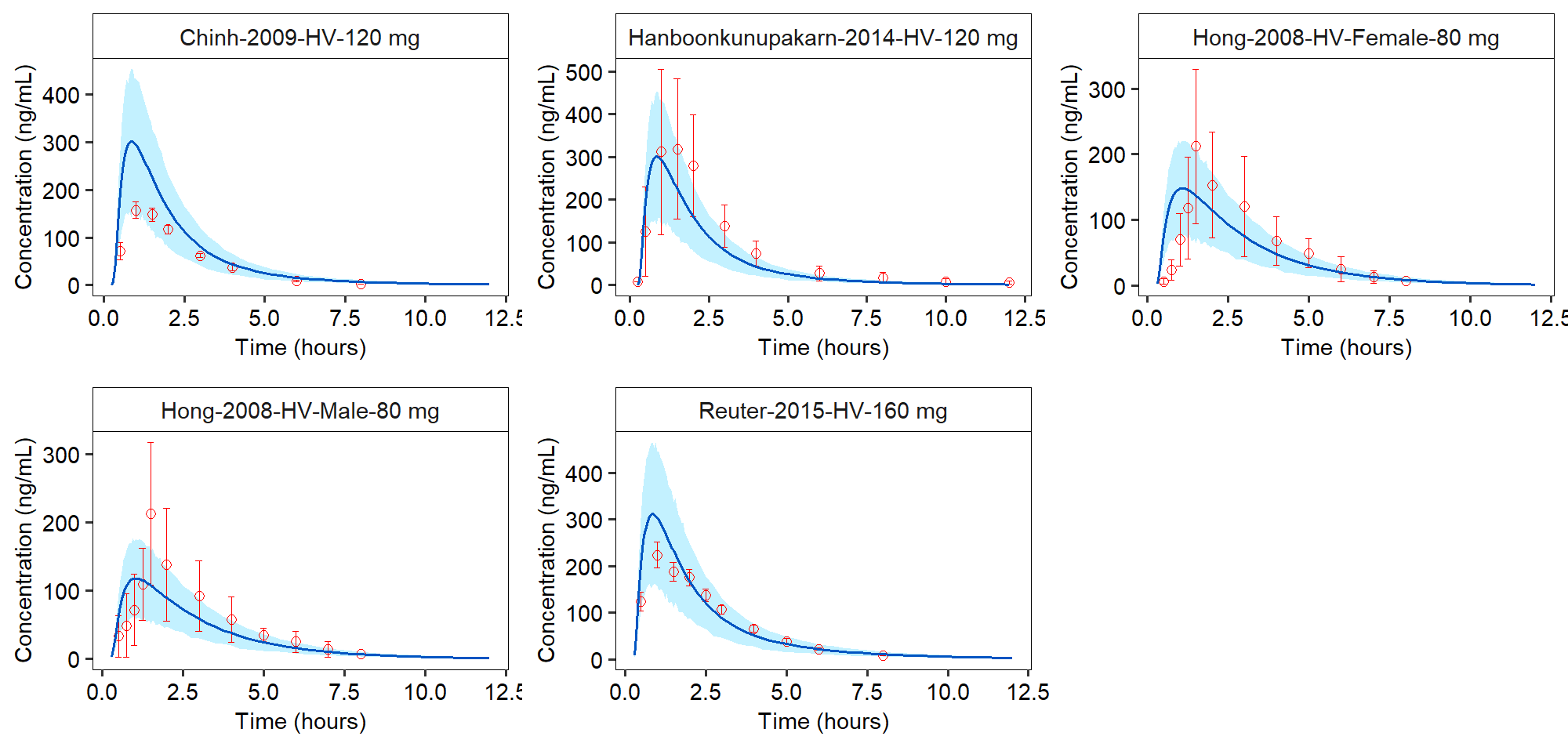
**

**Figure S2. PBPK prediction of dihydroartemisinin in a healthy population.**

The PBPK model incorporated key demographic elements (e.g., weight) aligned with the published studies. The blue line represents the predicted median concentration, while the shaded region depicts a 30% coefficient of variation (CV) around the predicted median. Red dots and error bars indicate observed average PK concentrations and their standard deviations.

a)


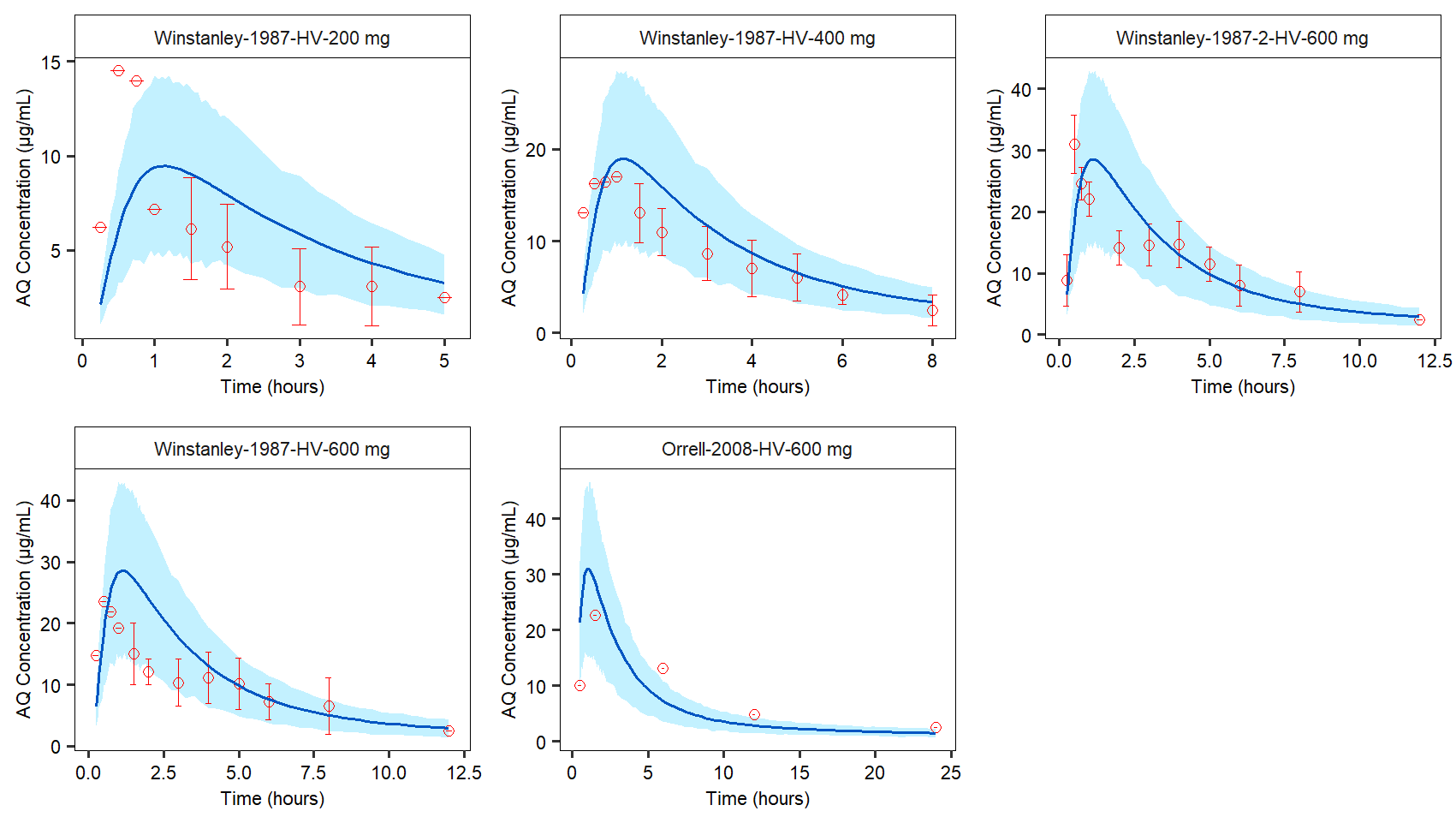


b)


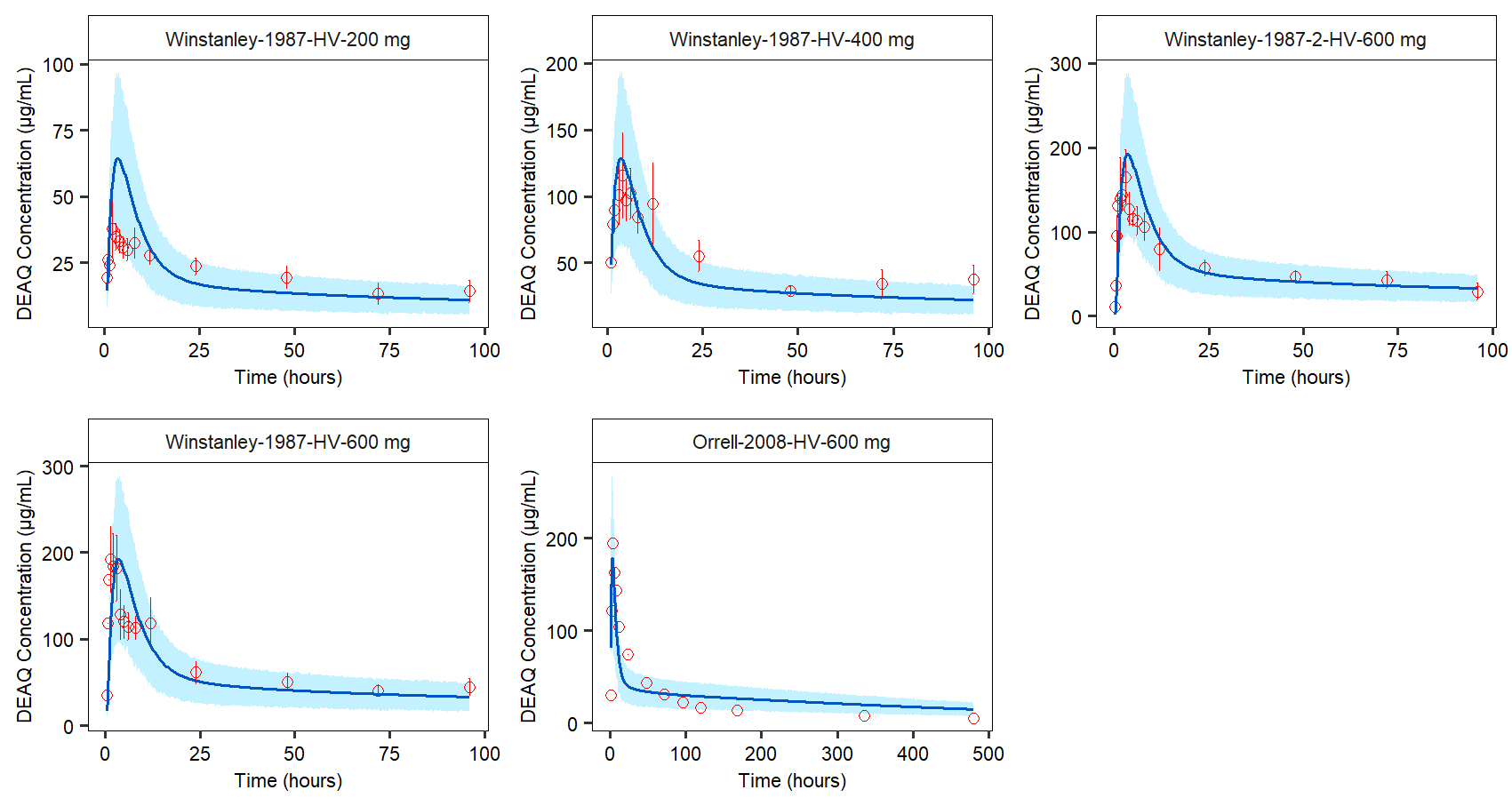


**Figure S3. PBPK prediction of amodiaquine (a) and desethylamodiaquine (b) in a healthy population.**

The PBPK model incorporated key demographic elements (e.g., weight) aligned with the published studies. The blue line represents the predicted median concentration, while the shaded region depicts a 30% coefficient of variation (CV) around the predicted median. Red dots and error bars indicate observed average PK concentrations and their standard deviations.


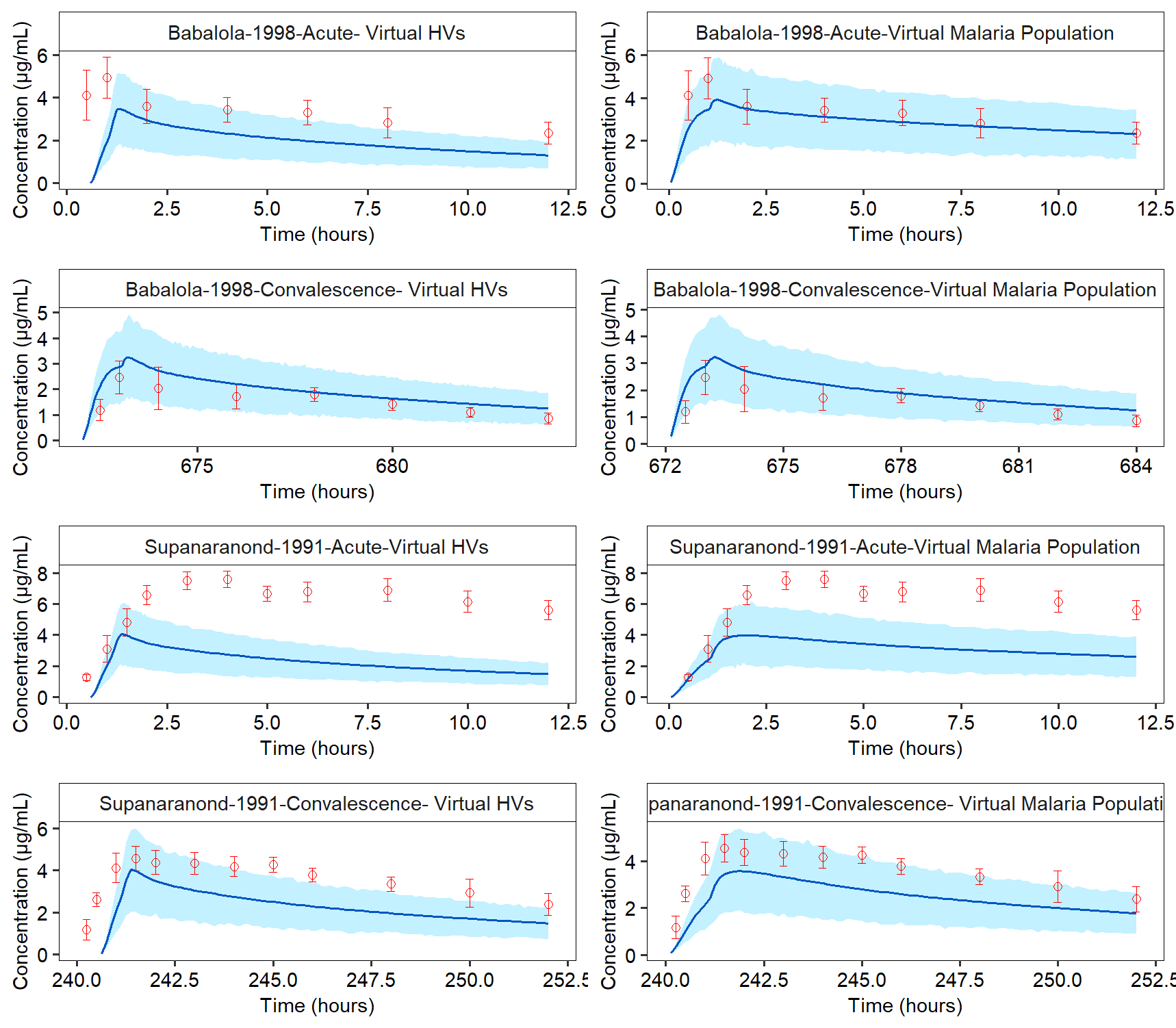


**Figure S4. PBPK prediction of quinine using a healthy volunteer population (left panel) and a virtual malaria population (right panel).**

The PBPK model incorporated key demographic elements (e.g., race, age, and weight) aligned with the published studies. The blue line represents the predicted median concentration, while the shaded region depicts a 30% coefficient of variation (CV) around the predicted median. Red dots and error bars indicate observed average PK concentrations and their standard deviations.

**
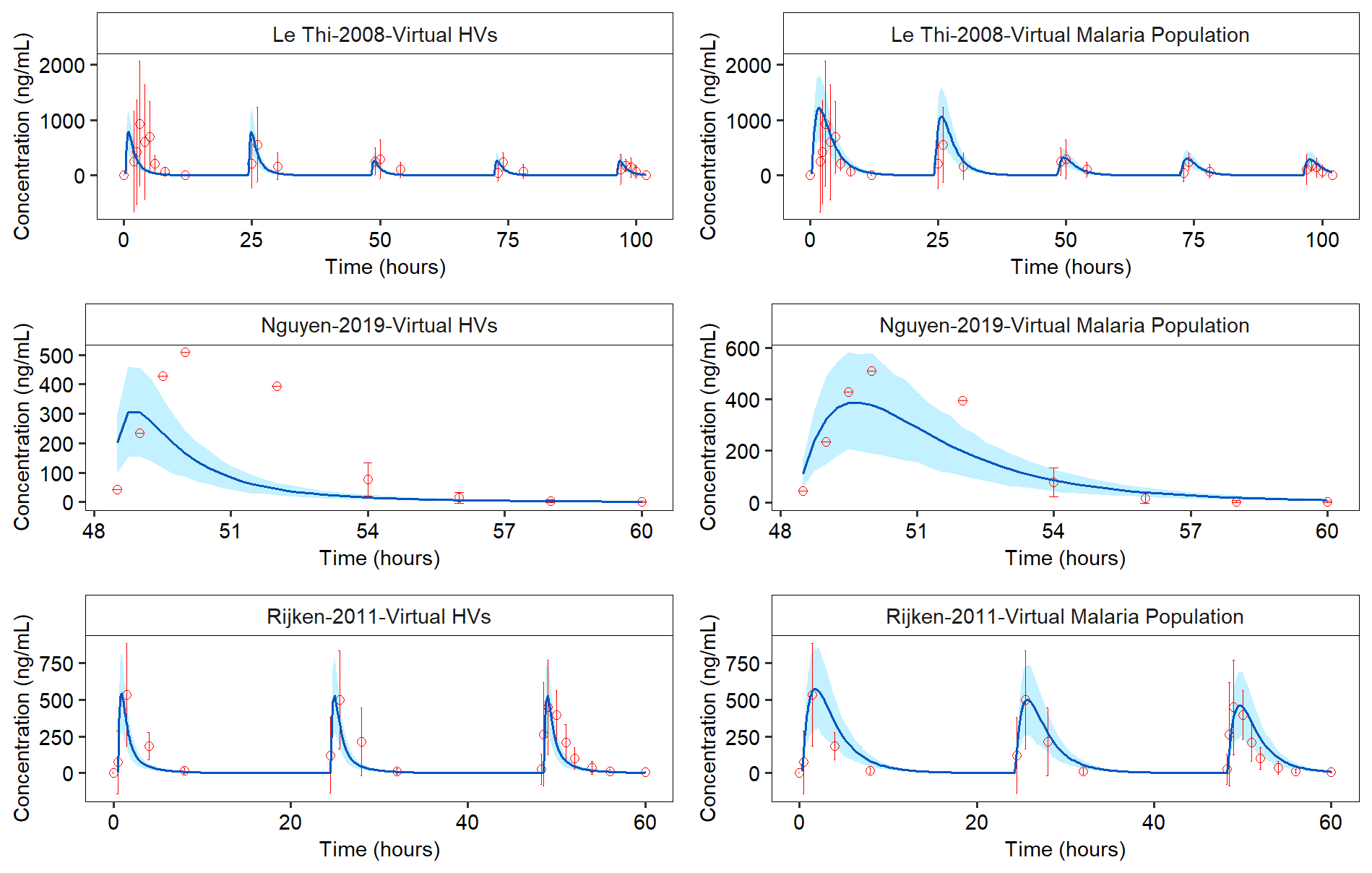
**

**Figure S5. PBPK prediction of dihydroartemisinin using virtual healthy (left panel) and malaria populations (right panel).**

The PBPK model incorporated key demographic elements (e.g., race, age, and weight) aligned with the published studies. The blue line represents the predicted median concentration, while the shaded


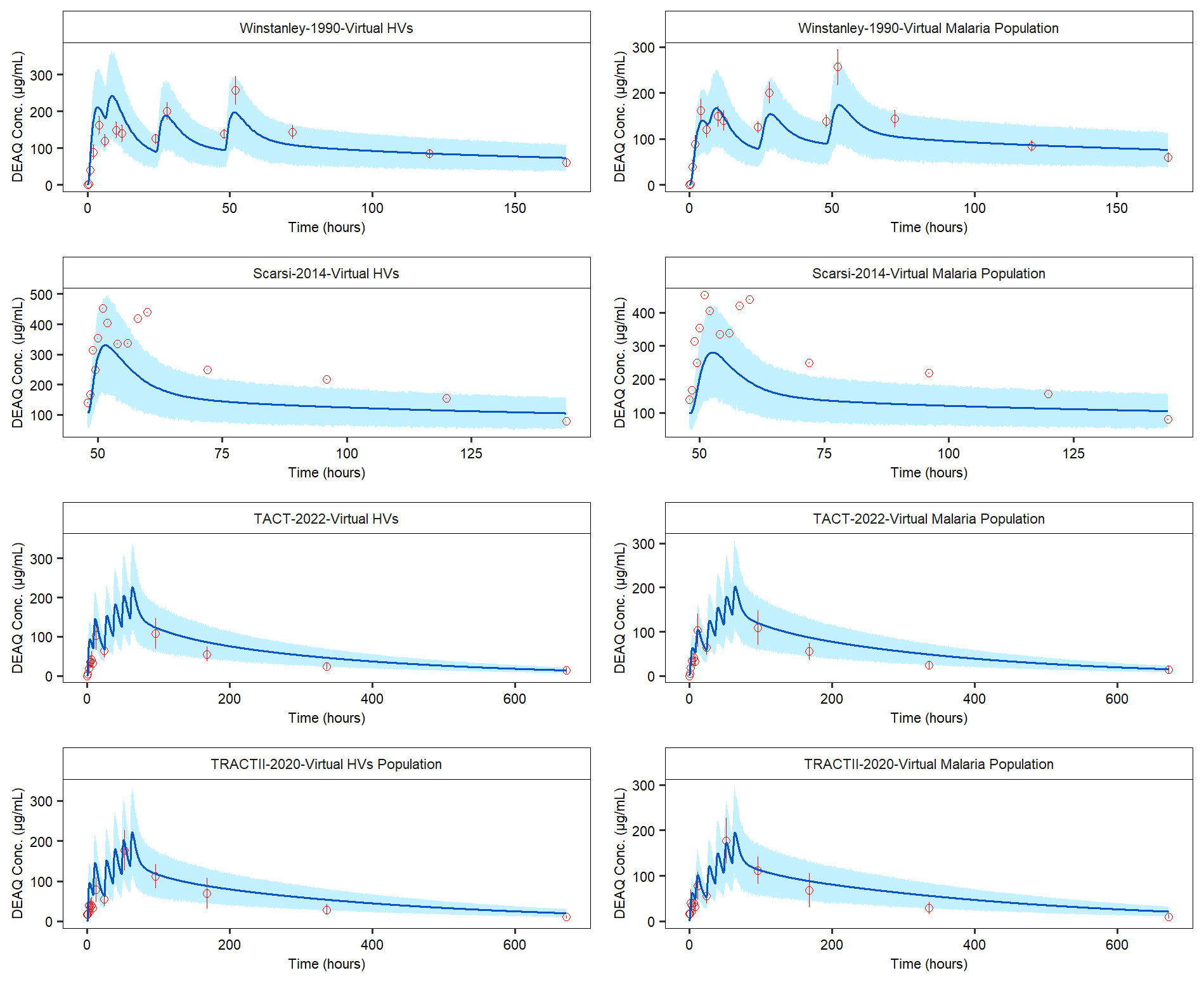

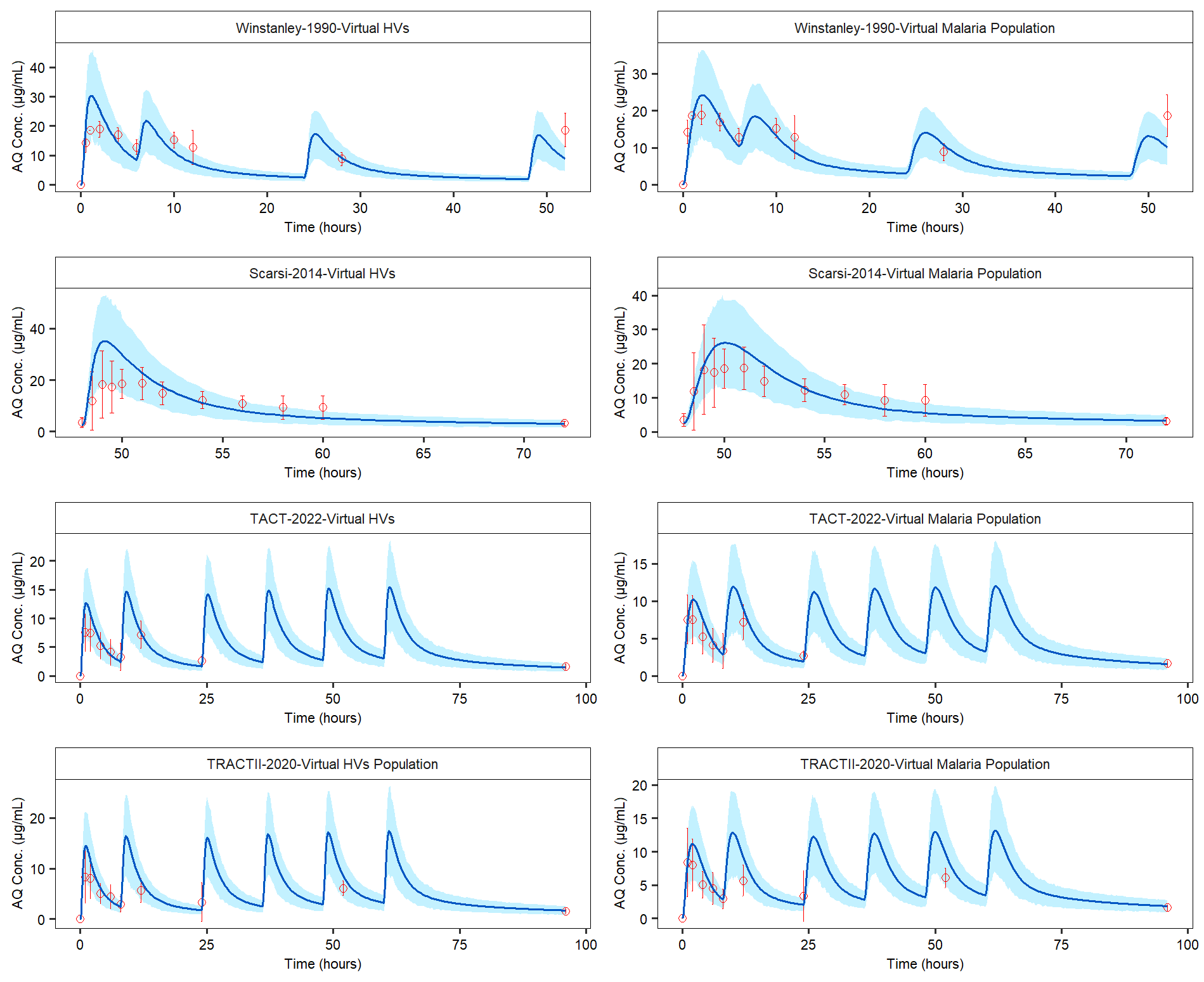


a)

b)

c)

**
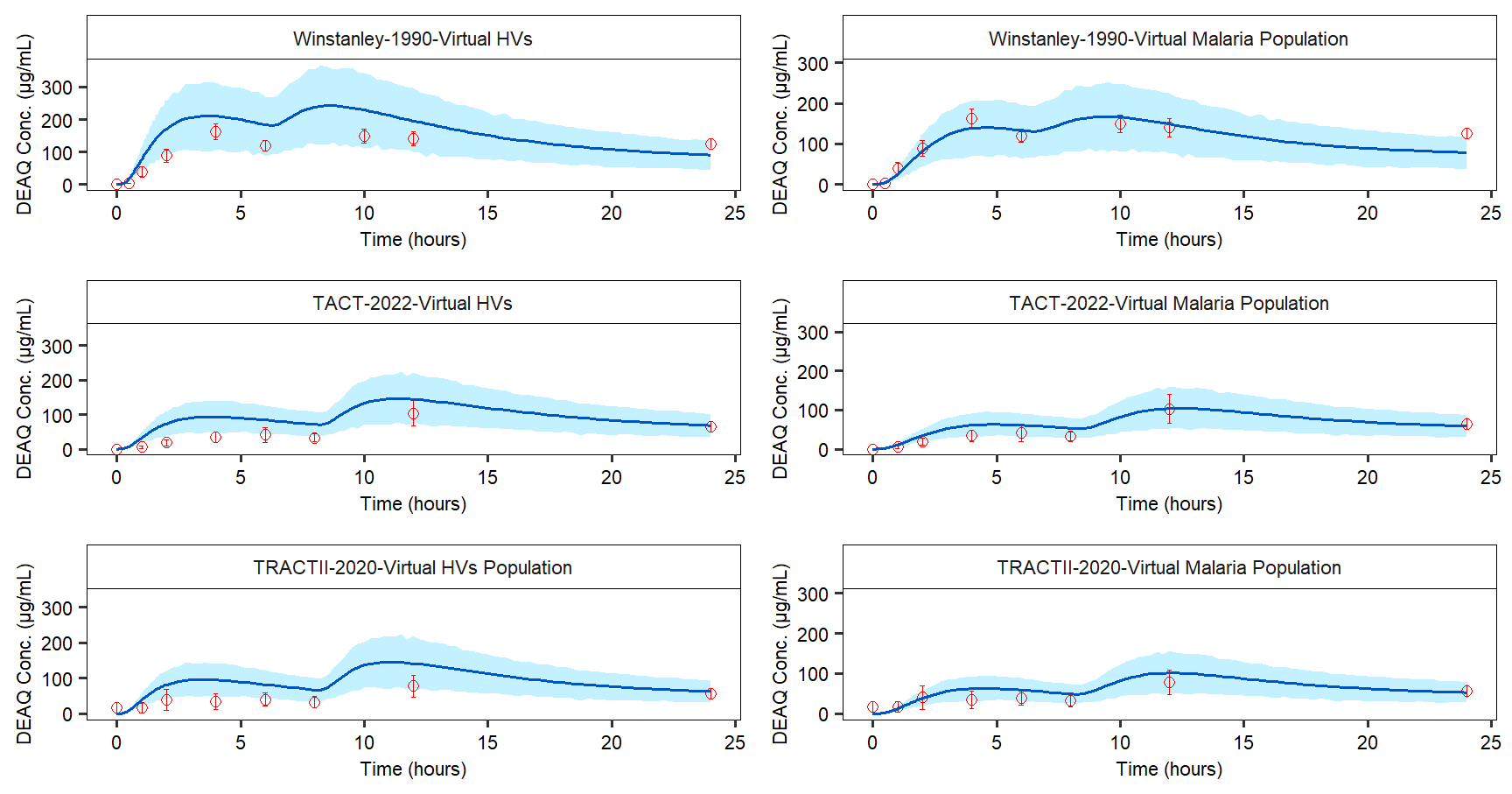
**

**Figure S6. PBPK prediction of amodiaquine (a) and desethyl-amodiaquine (b, c) using virtual healthy and malaria populations.**

AQ is amodiaquine, DEAQ is desethyl-amodiaquine. Panel C shows PK profile of DEAQ of the first 24 hours after the first dose. The PBPK model incorporated key demographic elements (e.g., race, age, and weight) aligned with the published studies. The blue line represents the predicted median concentration, while the shaded region depicts a 30% coefficient of variation (CV) around the predicted median. Red dots and error bars indicate observed average PK concentrations and their standard deviations.

**Supplementary References**

1. Winstanley PA, Edwards G, Orme ML, Breckenridge AM. Effect of dose size on amodiaquine pharmacokinetics after oral administration. Eur J Clin Pharmacol. 1987;33(3):331-3.

2. Winstanley P, Edwards G, Orme M, Breckenridge A. The disposition of amodiaquine in man after oral administration. Br J Clin Pharmacol. 1987;23(1):1-7.

3. Pussard E, Verdier F, Faurisson F, Scherrmann JM, Le Bras J, Blayo MC. Disposition of monodesethylamodiaquine after a single oral dose of amodiaquine and three regimens for prophylaxis against Plasmodium falciparum malaria. Eur J Clin Pharmacol. 1987;33(4):409-14.

4. Orrell C, Little F, Smith P, Folb P, Taylor W, Olliaro P, et al. Pharmacokinetics and tolerability of artesunate and amodiaquine alone and in combination in healthy volunteers. Eur J Clin Pharmacol. 2008;64(7):683-90.

5. Winstanley PA, Simooya O, Kofi-Ekue JM, Walker O, Salako LA, Edwards G, et al. The disposition of amodiaquine in Zambians and Nigerians with malaria. Br J Clin Pharmacol. 1990;29(6):695-701.

6. Scarsi KK, Fehintola FA, Ma Q, Aweeka FT, Darin KM, Morse GD, et al. Disposition of amodiaquine and desethylamodiaquine in HIV-infected Nigerian subjects on nevirapine-containing antiretroviral therapy. J Antimicrob Chemother. 2014;69(5):1370-6.

7. Peto TJ, Tripura R, Callery JJ, Lek D, Nghia HDT, Nguon C, et al. Triple therapy with artemether-lumefantrine plus amodiaquine versus artemether-lumefantrine alone for artemisinin-resistant, uncomplicated falciparum malaria: an open-label, randomised, multicentre trial. Lancet Infect Dis. 2022;22(6):867-78.

8. van der Pluijm RW, Tripura R, Hoglund RM, Pyae Phyo A, Lek D, Ul Islam A, et al. Triple artemisinin-based combination therapies versus artemisinin-based combination therapies for uncomplicated Plasmodium falciparum malaria: a multicentre, open-label, randomised clinical trial. Lancet. 2020;395(10233):1345-60.

9. Hong X, Liu CH, Huang XT, Huang TL, Ye SM, Ou WP, et al. Pharmacokinetics of dihydroartemisinin in Artekin tablets for single and repeated dosing in Chinese healthy volunteers. Biopharm Drug Dispos. 2008;29(4):237-44.

10. Chinh NT, Quang NN, Thanh NX, Dai B, Geue JP, Addison RS, et al. Pharmacokinetics and bioequivalence evaluation of two fixed-dose tablet formulations of dihydroartemisinin and piperaquine in Vietnamese subjects. Antimicrob Agents Chemother. 2009;53(2):828-31.

11. Hanboonkunupakarn B, Ashley EA, Jittamala P, Tarning J, Pukrittayakamee S, Hanpithakpong W, et al. Open-label crossover study of primaquine and dihydroartemisinin-piperaquine pharmacokinetics in healthy adult thai subjects. Antimicrob Agents Chemother. 2014;58(12):7340-6.

12. Reuter SE, Evans AM, Shakib S, Lungershausen Y, Francis B, Valentini G, et al. Effect of food on the pharmacokinetics of piperaquine and dihydroartemisinin. Clin Drug Investig. 2015;35(9):559-67.

13. Le Thi DT, Le NH, Nguyen CH, Phan Thi D, Na-Bangchang K. Pharmacokinetics of a five-day oral dihydroartemisinin monotherapy regimen in patients with uncomplicated falciparum malaria. Drug Metab Pharmacokinet. 2008;23(3):158-64.

14. Nguyen DV, Nguyen QP, Nguyen ND, Le TT, Nguyen TD, Dinh DN, et al. Pharmacokinetics and ex vivo pharmacodynamic antimalarial activity of dihydroartemisinin-piperaquine in patients with uncomplicated falciparum malaria in Vietnam. Antimicrob Agents Chemother. 2009;53(8):3534-7.

15. Rijken MJ, McGready R, Phyo AP, Lindegardh N, Tarning J, Laochan N, et al. Pharmacokinetics of dihydroartemisinin and piperaquine in pregnant and nonpregnant women with uncomplicated falciparum malaria. Antimicrob Agents Chemother. 2011;55(12):5500-6.

16. Karbwang J, Davis TM, Looareesuwan S, Molunto P, Bunnag D, White NJ. A comparison of the pharmacokinetic and pharmacodynamic properties of quinine and quinidine in healthy Thai males. Br J Clin Pharmacol. 1993;35(3):265-71.

17. Babalola CP, Bolaji OO, Ogunbona FA, Sowunmi A, Walker O. Dose linearity of quinine in healthy human subjects. European Journal of Pharmaceutics and Biopharmaceutics. 1997;44:143-7.

18. Igbinoba SI, Onyeji CO, Akanmu MA, Soyinka JO, Pullela SS, Cook JM, et al. Effect of dehusked Garcinia kola seeds on the overall pharmacokinetics of quinine in healthy Nigerian volunteers. J Clin Pharmacol. 2015;55(3):348-54.

19. Supanaranond W, Davis TM, Pukrittayakamee S, Silamut K, Karbwang J, Molunto P, et al. Disposition of oral quinine in acute falciparum malaria. Eur J Clin Pharmacol. 1991;40(1):49-52.

20. Babalola CP, Bolaji OO, Ogunbona FA, Sowunmi A, Walker O. Pharmacokinetics of quinine in African patients with acute falciparum malaria. Pharm World Sci. 1998;20(3):118-22.

21. Charman SA, Andreu A, Barker H, Blundell S, Campbell A, Campbell M, et al. An in vitro toolbox to accelerate anti-malarial drug discovery and development. Malar J. 2020;19(1):1.

22. Katneni K, Pham T, Saunders J, Chen G, Patil R, White KL, et al. Using Human Plasma as an Assay Medium in Caco-2 Studies Improves Mass Balance for Lipophilic Compounds. Pharm Res. 2018;35(11):210.

23. Mihaly GW, Ching MS, Klejn MB, Paull J, Smallwood RA. Differences in the binding of quinine and quinidine to plasma proteins. Br J Clin Pharmacol. 1987;24(6):769-74.

24. Allqvist A, Miura J, Bertilsson L, Mirghani RA. Inhibition of CYP3A4 and CYP3A5 catalyzed metabolism of alprazolam and quinine by ketoconazole as racemate and four different enantiomers. Eur J Clin Pharmacol. 2007;63(2):173-9.

25. Mirghani RA, Hellgren U, Westerberg PA, Ericsson O, Bertilsson L, Gustafsson LL. The roles of cytochrome P450 3A4 and 1A2 in the 3-hydroxylation of quinine in vivo. Clin Pharmacol Ther. 1999;66(5):454-60.

26. Batty KT, Ilett KF, Davis TM. Protein binding and alpha : beta anomer ratio of dihydroartemisinin in vivo. Br J Clin Pharmacol. 2004;57(4):529-33.

27. Na-Bangchang K, Krudsood S, Silachamroon U, Molunto P, Tasanor O, Chalermrut K, et al. The pharmacokinetics of oral dihydroartemisinin and artesunate in healthy Thai volunteers. Southeast Asian J Trop Med Public Health. 2004;35(3):575-82.

28. Senarathna SM, Page-Sharp M, Crowe A. The Interactions of P-Glycoprotein with Antimalarial Drugs, Including Substrate Affinity, Inhibition and Regulation. PLoS One. 2016;11(4):e0152677.

29. Li XQ, Bjorkman A, Andersson TB, Ridderstrom M, Masimirembwa CM. Amodiaquine clearance and its metabolism to N-desethylamodiaquine is mediated by CYP2C8: a new high affinity and turnover enzyme-specific probe substrate. J Pharmacol Exp Ther. 2002;300(2):399-407.

30. Akande A OS, Adebanjo AJ, Toyin AS, Ogbona OC. Effects of co-trimoxazole co-administration on the pharmacokinetics of amodiaquine in healthy volunteers. International Journal of Pharmacy and Pharmaceutical Sciences. 2015;7(9):272-6.

31. Winstanley PA, Edwards G, Curtis CG, Orme ML, Powell GM, Breckenridge AM. Tissue distribution and excretion of amodiaquine in the rat. J Pharm Pharmacol. 1988;40(5):343-9.

32. K Na-Bangchang SK, U Silachamroon, P Molunto, O Tasanor, K Chalermrut, N Tangpukdee, O Matangkasombut, S Kano, S Looareesuwan. Pharmaceutical Equivalence of Some Commercial Samples of Artesunate and Amodiaquine Tablets Sold in Southwestern Nigeria. Trop J Pharm Res. 2004;8(6):491.

33. SF H. Clinical pharmacokinetics and pharmacodynamics of antimalarial combination therapy: University of Gothenburg; 2009.

34. Zhang Y, Vermeulen NP, Commandeur JN. Characterization of human cytochrome P450 mediated bioactivation of amodiaquine and its major metabolite N-desethylamodiaquine. Br J Clin Pharmacol. 2017;83(3):572-83.
